# Near UV-Visible electronic absorption originating from charged amino acids in a monomeric protein†
†Electronic supplementary information (ESI) available. See DOI: 10.1039/c7sc00880e


**DOI:** 10.1039/c7sc00880e

**Published:** 2017-05-19

**Authors:** Saumya Prasad, Imon Mandal, Shubham Singh, Ashim Paul, Bhubaneswar Mandal, Ravindra Venkatramani, Rajaram Swaminathan

**Affiliations:** a Department of Biosciences and Bioengineering , Indian Institute of Technology Guwahati , Guwahati 781039 , Assam , India . Email: rsw@iitg.ernet.in; b Department of Chemical Sciences , Tata Institute of Fundamental Research , Homi Bhabha Road, Colaba , Mumbai 400005 , India . Email: ravi.venkatramani@tifr.res.in; c Department of Chemistry , Indian Institute of Technology Guwahati , Guwahati 781039 , Assam , India

## Abstract

Electronic absorption spectra of proteins are primarily characterized over the ultraviolet region (185–320 nm) of the electromagnetic spectrum. While recent studies on peptide aggregates have revealed absorption beyond 350 nm, monomeric proteins lacking aromatic amino acids, disulphide bonds, and active site prosthetic groups are expected to remain optically silent beyond 250 nm. Here, in a joint theoretical and experimental investigation, we report the distinctive UV-Vis absorption spectrum between 250 nm [*ε* = 7338 M^–1^ cm^–1^] and 800 nm [*ε* = 501 M^–1^ cm^–1^] in a synthetic 67 residue protein (α_3_C), in monomeric form, devoid of aromatic amino acids. Systematic control studies with high concentration non-aromatic amino acid solutions revealed significant absorption beyond 250 nm for charged amino acids which constitute over 50% of the sequence composition in α_3_C. Classical atomistic molecular dynamics (MD) simulations of α_3_C reveal dynamic interactions between multiple charged sidechains of Lys and Glu residues present in α_3_C. Time-dependent density functional theory calculations on charged amino acid residues sampled from the MD trajectories of α_3_C reveal that the distinctive absorption features of α_3_C may arise from two different types of charge transfer (CT) transitions involving spatially proximal Lys/Glu amino acids. Specifically, we show that the charged amino (NH_3_^+^)/carboxylate (COO^–^) groups of Lys/Glu sidechains act as electronic charge acceptors/donors for photoinduced electron transfer either from/to the polypeptide backbone or to each other. Further, the sensitivity of the CT spectra to close/far/intermediate range of encounters between sidechains of Lys/Glu owing to the three dimensional protein fold can create the long tail in the α_3_C absorption profile between 300 and 800 nm. Finally, we experimentally demonstrate the sensitivity of α_3_C absorption spectrum to temperature and pH-induced changes in protein structure. Taken together, our investigation significantly expands the pool of spectroscopically active biomolecular chromophores and adds an optical 250–800 nm spectral window, which we term ProCharTS (Protein Charge Transfer Spectra), for label free probes of biomolecular structure and dynamics.

## Abstract 2

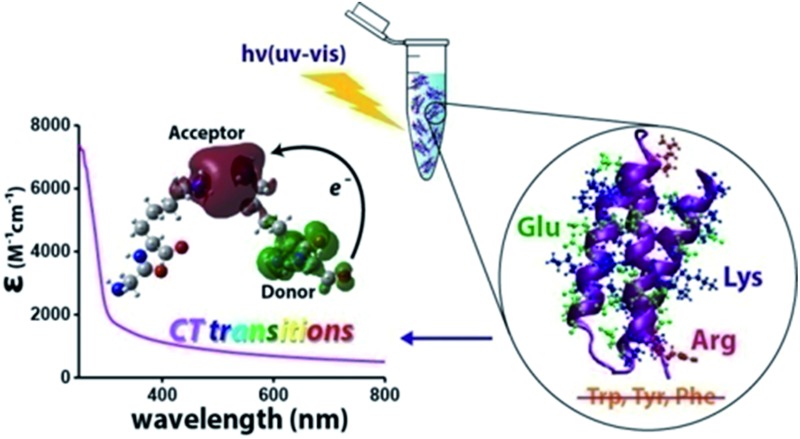
We report 250–800 nm UV-Vis monomeric protein absorption originating from protein backbone–sidechain and sidechain–sidechain charge transfer transitions involving Lys/Glu residues.

## Introduction

The characteristic electronic absorption profiles of proteins/amino acids in aqueous media show broad features in the UV region (185–320 nm) of the electromagnetic spectrum. The most distinctive absorption features of the spectra, typically seen around 255–280 nm, are attributed to chromophores present in the sidechains of aromatic amino acids like Trp around 280 nm (*ε* ∼ 5600 M^–1^ cm^–1^), Tyr around 275 nm (*ε* ∼ 1420 M^–1^ cm^–1^) and Phe around 257 nm (*ε* ∼ 197 M^–1^ cm^–1^). Weak contribution from disulphide bonds between 250 nm (*ε* ∼ 360 M^–1^ cm^–1^) to 320 nm (*ε* ∼ 6 M^–1^ cm^–1^) has also been reported.^1^–^3^ The peptide bond in proteins has a strong absorption around 190 nm (*ε* ∼ 7000 M^–1^ cm^–1^) and a weak absorption between 210 and 220 nm (*ε* ∼ 100 M^–1^ cm^–1^).^4^–^7^ Further, chromophores present in prosthetic groups and metal–ligand centres localized at enzyme active sites absorb in the visible (beyond 400 nm).^2^ Accordingly, proteins devoid of aromatic amino acids, disulphide bonds, and active-site chromophores are expected to remain optically silent at wavelengths beyond 250 nm.^8^,^9^ Several years ago, our group had reported significant absorption in the 250–350 nm region for poly-l-Lys solutions and in Lys-rich proteins such as human serum albumin (HSA) after accounting for the absorption from dominant chromophores like Trp.^10^ Interestingly, the UV absorption band beyond 320 nm is absent in many proteins, particularly those that have low Lys content.^10^ Further, novel absorption spectra (*λ*_max_ ∼ 270 nm) were also seen in concentrated (0.5–1.0 M) pure aqueous solutions of l-Lys mono-hydrochloride (Lys·HCl),^11^ and corroborated by other researchers later.^12^ The results above are intriguing because Lys has an aliphatic sidechain ending with a primary amine that cannot possibly absorb in the near-UV region in its monomeric form. Recently, several investigators have also reported unusual UV-Vis absorption/visible fluorescence beyond 350 nm from protein powders, high concentration protein solutions, and peptide aggregates lacking aromatic amino acids.^13^–^17^ The suggested mechanisms for the unusual absorption and fluorescence include backbone H-bonding and proton transfer. Our previous studies suggest that proteins rich in charged amino acids may also absorb between 250 and 350 nm even if they lack aromatic amino acids.^10^ Establishing a quantitative link between non-aromatic protein amino acid sequence content and the UV-Vis absorption features above 320 nm would open up a new spectral window to probe prominent proteins of biomedical relevance.^18^–^21^ In particular, charged amino acids Lys and Arg are integral constituents of DNA and RNA binding proteins such as histones, spliceosomal proteins and transcription factors. His is an important constituent of glycoproteins.^22^ Glu rich proteins play important roles in estrogen receptor binding.^23^,^24^ Finally, intrinsically disordered proteins which play a key role in several regulatory events inside the human cell are rich in charged amino acids.^25^ These considerations motivate a systematic investigation of protein absorption spectra beyond 320 nm and its dependence on charged amino acid content. Given the strong prominent spectral features of Trp, Tyr and Phe, it is desirable to investigate the UV-Vis absorption spectra within a model protein devoid of these aromatic amino acids.

**Fig. 1:**
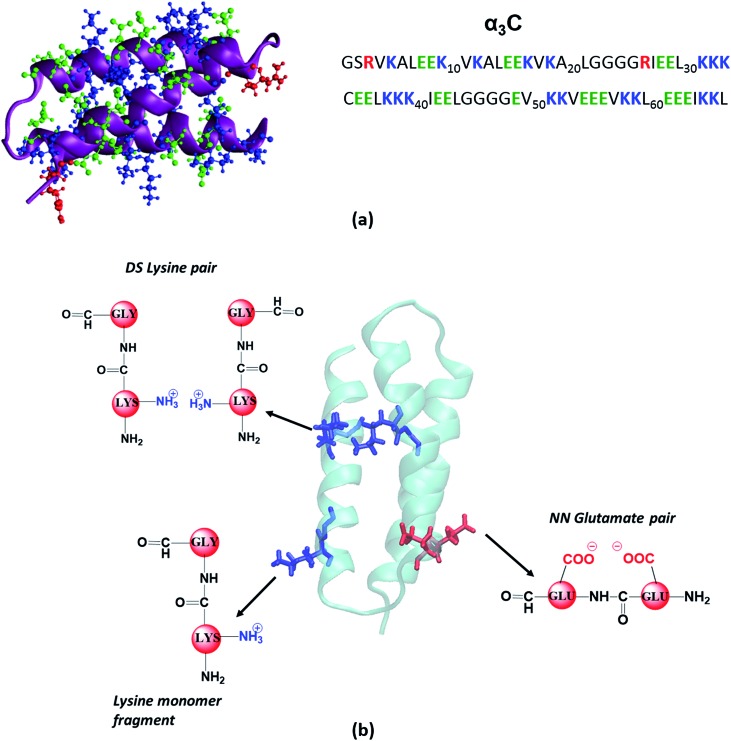
(a) Structure (PDB code: 2LXY) and amino acid sequence of α_3_C protein; (b) examples of fragment models extracted from classical MD generated structures of α_3_C. For monomer models we extracted a dimer containing Lys/Glu/Gly, mutated the other amino acid to Gly and capped the amino and carbonyl ends with hydrogens. The same procedure was followed for each monomer in Distally Separated (DS) in sequence pairs. Nearest Neighbour (NN) in sequence residue pair dimers were directly capped with hydrogens.

In this study, we carried out systematic experimental and theoretical investigations on the UV-Vis absorption spectrum (250–800 nm) of a small (67 residue), monomeric, synthetic protein devoid of aromatic amino acids (α_3_C). The structure of α_3_C has been determined previously by NMR experiments^26^ to be a three helix bundle (Fig. 1a). The protein is rich in charged amino acids (54% of the sequence) which comprise of 17 Lys, 17 Glu, and 2 Arg residues (Fig. 1a). Despite lacking conventional aromatic chromophores, α_3_C exhibited moderate absorption features in the 250–320 nm region (*ε* = 7338 ± 191 M^–1^ cm^–1^ at 250 nm) and a distinctive long tail spanning the entire visible spectrum up to 800 nm (*ε* = 964 ± 129 and 501 ± 66 M^–1^ cm^–1^ at 450 and 800 nm, respectively). We carried out control absorption spectra measurements in high concentration aqueous solutions of all non-aromatic amino acids including those present in α_3_C and confirmed that charged amino acids possess unique characteristic absorption features extending beyond 320 nm. Time-dependent density functional theory (TDDFT) electronic structure calculations on amino acids of the α_3_C protein sampled from classical molecular dynamics (MD) trajectories revealed that Lys and Glu amino acids may produce broad absorption spectral profiles. Analysis of the computed spectra showed that Lys and Glu amino acids possess charge transfer (CT) transitions involving the amino (NH_3_^+^)/carboxylate (COO^–^) groups of their sidechains and the polypeptide backbone. Our MD simulations further highlighted the spatially proximal (4–6 Å) interactions between charged amino/carboxylate groups of Lys/Glu sidechains facilitated by the three dimensional (3D) protein fold. We show that such spatial interactions between charged residues can modulate the spectral transitions above 300 nm and create a long tail in the α_3_C absorption spectra. Finally, we attempt to connect the experimentally observed changes in the α_3_C absorption spectra triggered by changes in solvent pH and temperature with alterations in the separation between charged amino (Lys) and carboxylate groups (Glu) of α_3_C in the 3D space.

We term the new 250–800 nm absorption spectra from CT transitions in charged amino acids as ProCharTS (Protein Charge Transfer Spectra). ProCharTS provides new label free spectral markers to track the structure and dynamics of proteins rich in charged amino acids which can complement traditional techniques based on aromatic chromophores. In the manuscript, we provide evidence for ProCharTS spectral signatures in proteins containing aromatic amino acids. Thus, the method is generally applicable to study natural proteins or protein domains which are rich in charged amino acids irrespective of their aromatic amino acid content.

## Methods

### Search and selection of α_3_C for experimental and computational studies

We scanned all available PDB codes from RCSB Protein Data Bank (http://www.rcsb.org) and corresponding FASTA sequence to examine their charged amino acid content. Based on our exhaustive search, 2-mercaptoethanol-α_3_C protein (PDB ID: ; 2LXY), was selected for further studies based on high content and close proximity of Lys residues. The α_3_C protein (Fig. 1a) contains 17 Lys residues. Out of these, 14 Lys pairs are within 10 Å distance. More details on this search and selection procedure is presented in the ESI (Section S1.1).†

### Experimental methods

All the amino acids and the control protein samples of the highest purity available were purchased from Sigma Aldrich Chemicals Pvt. Limited, Bengaluru, India. Control proteins were Human Serum Albumin (HSA; cat #A1887) and Hen Eggwhite Lysozyme (HEWL; cat #L6876). Other chemicals and reagents of high purity analytical grade were procured from Merck India Limited.

#### Expression and purification of α_3_C

The recombinant α_3_C was over expressed in *E. coli* and purified as per the methodology described elsewhere.^26^ The purification of protein was monitored (see Section S1.2 of ESI†) at each step by SDS-PAGE (Fig. S1a of ESI†). Purity was further confirmed by reverse phase HPLC (Fig. S1b of ESI†) and the mass ascertained by Electrospray Mass Spectrometry to be 7462.883 Da (Fig. S1c of ESI†). The lyophilized sample of the protein was used for further experiments.

#### Solid phase peptide synthesis of peptides containing Lys

Peptides with varying distance between the Lys residues (NH_2_–Gly–Lys–Lys–Gly–CONH_2_, NH_2_–Gly–Lys–Ala–Lys–Gly–CONH_2_, NH_2_–Gly–Lys–Ala–Ala–Lys–Gly–CONH_2_) were synthesized by standard Fmoc/tertiary-butyl orthogonal protection strategy using solid phase peptide synthesis. The syntheses were performed manually on a Stuart blood tube rotator. Peptides were synthesized such that each peptide had two Lys residues with variable separation in sequence. The steps involved in the synthesis and characterization are described in detail in the Sections S1.3 and S1.4 of ESI.† Unless stated specifically, all reactions were carried out at room temperature.

#### UV-Visible absorption spectra

The absorption spectra for all non-aromatic amino acids, Lys containing peptides, and poly-l-Lys were recorded at room temperature (25 °C) on a double beam Lambda-25 UV-Vis Spectrophotometer (Perkin Elmer, USA) using a UV quartz cell of 10 mm path length. Flatness of baseline was ascertained before and after all measurements by running the blank solution in sample and reference cuvette chambers. Spectra were acquired with multiple scans (3–5) between 250 and 800 nm (fixed 1 nm bandwidth) and averaged subsequently. For recording temperature dependent spectra at 25 °C and 85 °C, Varian Cary-100 double beam spectrophotometer equipped with a Peltier-based sample temperature controller was used. The sample was thermally equilibrated at high temperature for at least 30 minutes prior to recording absorption spectra.

The Lys containing peptides and Poly-l-Lys·HCl samples were dissolved in deionized water. The amino acids, *viz.* Ala, Arg, Glu·Na, Asp·K, Gly, Lys, Lys·HCl, Pro, and Ser were dissolved in deionized water, while Asn, Cys, His, Ile, Leu, Thr and Val were dissolved in 0.1 N HCl as they were insoluble in pure water. For all the scans, 1 M concentrations were employed unless otherwise stated. Control studies of pH dependence of Lys solutions are provided in the ESI (Fig. S4).†

The α_3_C protein was dissolved in deionized water and the absorption spectra (200–800 nm) were recorded for different concentrations (5–105 μM) of the protein. Pure deionized water was kept as blank control for the measurements. Protein concentrations were calculated using the Lowry method and confirmed by measuring the difference in far UV absorbance (*A*_215_ – *A*_225_).^27^,^28^ We carried out pH dependent studies on α_3_C (85 μM) dissolved in pure deionized water by gradual addition of either 0.1 N NaOH or 0.1 N HCl to the protein solution. Absorption spectra from 250 to 800 nm were recorded as stated earlier.

#### Circular dichroism measurements

CD measurements were carried out on α_3_C at 25 °C and 85 °C on a spectropolarimeter (Make: JASCO, Model: J-1500, JASCO Inc., Maryland, USA). The scans were recorded from 300 to 190 nm with data pitch of 0.1 nm, bandwidth of 2 nm; thinning scale was kept at 9 and the dynode voltage never exceeded 0.6 kV. Three scans were recorded for each sample and deionized water served as blank in all the cases. Quartz Cuvette (Make: JASCO) with 1 mm path length with transmission range up to 190 nm was used for recording all the measurements.

### Computational methods

#### Molecular dynamics (MD) simulations of α_3_C

We carried out MD simulations on fully solvated atomistic models of the α_3_C protein using the NAMD program^29^ (version 2.9) and the CHARMM27 force field.^30^ The initial structure used in the simulations was an NMR derived structure (PDB code: ; 2LXY) captured with mercaptophenol ligated at the C32 site. The ligand was removed during processing to carry out simulations of mercaptophenol free α_3_C. The Protein Data Bank (PDB) structures had 31 frames and we chose frame 15 (this frame had the maximum number of Lys residues within 10 Å of each other) as the reference structure for simulations. First hydrogens were added to the structure using the psfgen utility in the VMD program^31^ and the protein was solvated (TIP3P water model) inside a rectangular water box of dimensions ∼67 × 56 × 60 Å^3^. The volume for a single protein molecule mimics a protein solution of ∼7.4 mM concentration. This is worked out as follows: a water box of volume 67 × 56 × 60 Å^3^ = 225 120 × 10^–30^ m^3^ solution contains 1 protein molecule. This implies that 1000 litre (1 m^3^) of water contains 1/(225 120 × 10^–30^) molecules. Thus, 1 litre of water contains 1/(225 120 × 10^–30^ × 1000 × 6.022 × 10^23^) mol = 7.38 × 10^–3^ mol of protein. The system was neutralized by adding 2 Cl^–^ ions. Following standard equilibration protocols (see Section S1.5 of ESI†) a 110 ns MD NPT production run was carried out generating snapshots at interval of 2 ps. The α_3_C protein structure was found to be stable in the 3-helix bundle form along the trajectory. Data from the last 100 ns of the MD production run was used for analysis shown in the manuscript.

#### Electronic structure calculations

We computed the absorption spectra of amino acid fragments (see below) extracted from 100 α_3_C snapshot structures sampled from the last 100 ns of the MD production run (see procedure below and Fig. 1b). For 100 conformations of each amino acid fragment listed below, absorption spectra were calculated using TDDFT with a CAM-B3LYP^32^ functional and the 6-31++G(d) basis set on all atoms in the Gaussian 09 program.^33^ In all TDDFT calculations, the first 100 to 200 lowest energy electronic transitions were calculated. Since some systems exhibited transitions deep in the visible range of wavelengths, more than 100 transitions were required to cover the absorption spectral range. Difference electron density plots were calculated using the Multiwfn 3.3.8 software^34^ and visualised (isovalue set to 0.0004) in GaussView 5.0.^35^

#### Calculations of monomer, dimer, and peptide spectra

The amino acid fragments included monomers (Gly, Lys, and Glu), dimers (Lys–Lys, Glu–Glu, Lys–Glu, Lys–Ala, Lys–Ile, Lys–Leu, Lys–Val, Lys–Cys), pairs of dimers (Lys:Gly–Lys:Gly, Glu:Gly–Glu:Gly, Lys:Gly–Glu:Gly), and tetramers (Gly–Gly–Gly–Gly). The dimers and dimer pairs represent models for interactions among nearest neighbour (NN) and distally separated (DS) amino acid pairs in sequence (data in Fig. 6 and S13 of ESI†). For each amino acid fragment extracted from the trajectory, dangling bonds were capped using the psfgen module in VMD with modified C terminus (CHO group) and N terminus (NH_2_ group). NN interacting pair dimer fragments (data in Fig. S13 of ESI†) and Gly tetramers (data in Fig. S5 and S6 of ESI†) were capped directly. However, for monomers (data in Fig. 4) and DS pairs (data in Fig. 6), we capped the charged amino acid backbones with Gly to better represent the polypeptide backbone in the protein environment. The procedure involved extraction of Lys/Glu and its adjacent residue and then mutating the adjacent residue in the fragment to Gly. For DS pairs the capping procedure described above was applied to both charged amino acids of the pair (Fig. 1). NN and DS pairs of charged amino acid residues were chosen based on the distance of amino nitrogen (N_A_) and carboxylate carbon (C_C_) atoms of their sidechain. Three cases of (1) strong, (2) intermediate, and (3) weak interactions were considered based on distance ranges extracted from the RDF plots for Lys N_A_ and Glu C_C_ atoms (data in Fig. 5b).

#### Spectra calculations modelling the effects of the environment

For selected amino acid fragments described above we re-computed the TDDFT spectra after including explicit water molecules and other charged chemical species in the vicinity of the fragment representative of the polar environment of the protein surface. The following procedure was used to construct these models: for Lys, Glu monomers and DS Lys–Lys, Glu–Glu and Lys–Glu dimer pairs, 10 MD snapshots (from within the 100 used for vacuum calculations) were chosen. We included explicit waters and/or Glu carboxylate groups within 3–6 Å from either the Lys N_A_ or Glu C_C_ atoms (monomers) or the geometric centre of interacting N_A_ and C_C_ atoms (dimers). For the case of Lys–Lys, and Lys–Glu dimer pairs we also examined the effect of water position (the electronic coupling effect of the water to the dimer pair) on the spectra by manually placing the water molecule at different distances from the charged amino/carboxylate groups. In order to examine the effect of changes in protonation states of interacting dimers, we selected single representative MD snapshots of DS Lys–Lys and Lys–Glu dimer pairs and recomputed the TDDFT spectra after either deleting H atoms from the NH_3_^+^ groups or adding H to the COO^–^ groups. We covered all 3 possible deprotonation sites for the amino group N_A_ atoms and the 2 possible protonation sites for the carboxylate group O atoms.

#### Characterization of transitions

Two measures were used to characterize the transitions as charge transfer (CT) transitions or non-CT transitions. The first measure is the average hole–electron separation distance, Δ*r*:^34^,^36^
1
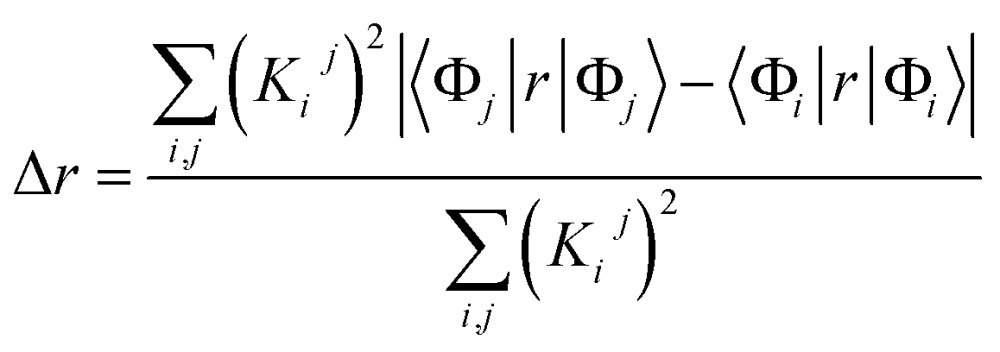
where Φ is the molecular orbital (MO) and the index *i* and *j* go over all occupied and vacant MOs respectively. Here *K*_*i*_^*j*^ = *P*_*i*_^*j*^ + *Q*_*i*_^*j*^ where *P*_*i*_^*j*^ and *Q*_*i*_^*j*^ are excitation (*i* → *j*) and de-excitation (*j* ← *i*) configuration coefficients. The second measure is the distance between the centroid of the hole and electron distribution (*D*_CT_), defined as:^34^,^37^
2
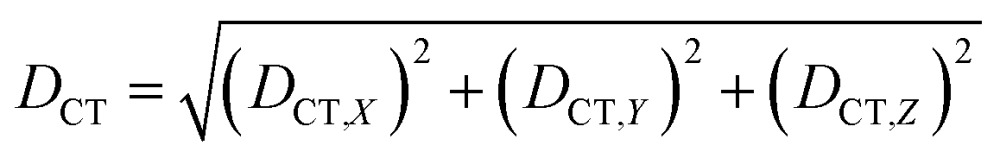
with3
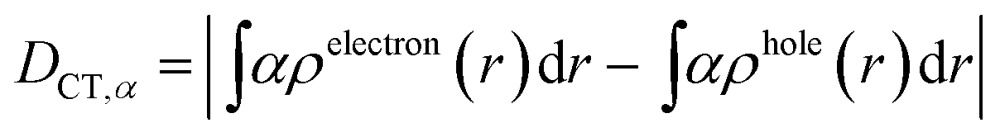
where, the index *α* represents Cartesian components (*X*, *Y*, *Z*) and *ρ*^electron/hole^ is the electron/hole density distribution. The two measures were calculated with Multiwfn version 3.3.8 34 and then the following conditions were used to categorize the transitions: (i) CT transition when Δ*r* > 2 Å and *D*_CT_ > 1 Å, (ii) non-CT transition, when Δ*r* = <2 Å or if Δ*r* > 2 Å and *D*_CT_ < 1 Å. Depending on the overlap integral between the hole and electron distribution, non-CT transitions can be further classified into: (1) local excitations: large overlap integral values, and (2) Rydberg transitions: small overlap integral values. In our analysis (data in Fig. 4 and S15 of ESI†), we simply classify the transitions as CT and non-CT transitions. Further, transitions for which the Δ*r* was within 5% of the threshold (2 Å) value were classified as borderline transitions.

#### Critical discussion of modelling methods and assumptions

In our computational protocol we sampled chromophore (amino acid monomers and interacting dimers) conformations from fully solvated and atomistic MD simulation trajectories of the α_3_C protein. Thus chromophore configurations used in the TDDFT calculations are completely compatible with the solution phase measurements. The objective of our computational studies in this manuscript is to examine the nature of transitions in charged amino acids and the modulation of their spectral range with conformational fluctuations and side-chain associations. To this end, we carried out electronic structure calculations on more than 2500 conformations (each panel of spectral profiles in Fig. 4, 6, and associated ESI† shows data from 100 or more structures) of relevant amino acid chromophores sampled from the MD simulations. The two central conclusions from our computational studies relate to physical properties of the chromophores: (1) charged amino acids possess intrinsic CT transitions due to the directional electric field created by the excess charge on their sidechains, and (2) interactions between the charged sidechains alter the nature of charge donor–acceptor states to modulate the absorption spectral range of such CT transitions. These conclusions are further supported by our control experimental data comparing the spectra of charged *vs.* non-charged amino acids (data in Fig. 3).

We critically examined capping strategies for amino acids in our electronic structure calculations using control calculations on extended peptide backbones and alternative capping models using methyl groups^38^ (Fig. S5–S7 of ESI†). Based on these calculations, we concluded that extending the polypeptide backbone by adding an extra Gly unit to the C-terminal end of amino acid fragments provided robust converged spectra above 250 nm. Our control calculations show that further extending the backbone by adding Gly units or using methyl capping groups (Fig. S5 and S6 of ESI†) only alters backbone transitions around 200 nm. However, backbone transitions and signature CT transitions involving charged amino acid sidechains/backbone near and above 250 nm are not altered by either extending the backbone or changing the capping groups. We do find a small number of fictitious non-CT transitions above 300 nm localized on our capping groups (Fig. 4 and S7 of ESI†). However, we were able to cleanly identify and separate these non-CT transitions above 300 nm from our characteristic CT transitions of interest (see Fig. 4 and S15†).

We have chosen the TDDFT method to compute the UV-Vis spectral profile for the amino acid chromophores. The method scales well with system size and has been shown to provide reasonable results for simulating UV-Vis spectra of organic chromophores and amino acids.^17^,^39^–^41^ We employed a range corrected exchange-correlation (XC) functional (CAM-B3LYP) which provides a reasonable description of charge transfer excitations and UV-Vis spectra in dipeptides and tripeptides.^32^,^42^

Environmental/solvation effects are typically included in spectra calculations through continuum dielectric or quantum mechanics (QM)/molecular mechanics (MM) models.^40^,^43^–^45^ These models are best suited for the description of isolated (spectrally distinct) chromophores (*e.g.* dyes or aromatic amino acids) embedded in a solvent and/or protein medium. Even in such situations, it is advisable to use continuum solvation models coupled with an explicit QM description of solvent molecules interacting with the solute.^40^,^46^ Depending on the nature of the transitions, hundreds of solvent molecules may be required to converge spectral trends.^44^,^46^ A reasonable criterion for choosing the size of the QM region is to include all residues/solvent molecules whose charge distribution changes significantly during the chemical reaction of interest.^47^ In other words, the MM region should only include those molecules whose charge distribution is fixed during the reaction. Our system shows highly anisotropic solvation with concentrated charged moieties dynamically interacting on the surface of a protein along with bound waters. Here, during photoexcitation, all charged amino acids (and maybe even bound waters) will show significant changes in their charge distribution (since the reaction involves CT transitions). Thus, the choice of the QM/MM boundary is non-trivial in our case. To examine environmental effects, a rational way forward is to systematically increase the size of the QM region accompanied by sampling configurations with different charge states. Thus, in our TDDFT spectra calculations, we focus on developing explicit QM models to describe environmental effects. Specifically, we considered the effect of the polar protein surface on the spectral features by examining different charged sidechain states and by including explicit water molecules and charged sidechains in the vicinity of the chromophores.

## Results and discussion

### UV-Vis spectra of α_3_C reveals significant absorption spanning 250–800 nm

**Fig. 2:**
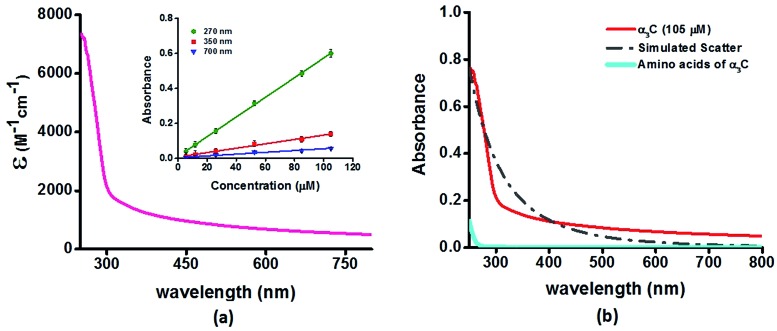
(a) Absorption spectrum (250–800 nm) of α_3_C in water. Inset shows the concentration dependence of absorbance at different wavelengths; (b) comparison of absorption spectrum (250–800 nm) of the intact α_3_C protein with that of a solution mixture of constituent amino acids in α_3_C (sequence shown in Fig. 1a). The relative concentration of amino acids in the mixture (data provided in Table S2†) was chosen based on their proportions present in a 105 μM solution of α_3_C. Simulated scatter is shown as dots and dashes. In Fig. S3 of ESI† we show an extended absorption spectrum (200–800 nm) to highlight the backbone contribution near 200 nm.

We investigated the UV-Vis spectra between 250 and 800 nm for different solution concentrations of α_3_C ranging from 5 to 105 μM. The molar extinction coefficient (Fig. 2a) reveals moderate absorption (*ε* = 7338 ± 191 M^–1^ cm^–1^ at 250 nm) features in the 250–300 nm region which decay gradually with a distinctive long tail that extends into the visible region (*ε* = 964 ± 129 and 501 ± 66 M^–1^ cm^–1^ at 450 and 800 nm, respectively). The observed spectral features (the tail region) are clearly not due to scattering, as demonstrated by the poor overlap of the observed spectra with a simulated Rayleigh scattering profile which follows a (1/*λ*^4^) dependence (Fig. 2b). Further, the absorbance at different wavelengths varies linearly with concentration (Fig. 2a-inset), arguing against any contribution arising from protein intermolecular interactions to the spectra. Indeed, the monomer proteins are likely to be farther than 20 nm from each other, on average, at 105 μM concentration. While absorption above 320 nm was seen previously in proteins rich in charged amino acids such as HSA (*ε* = 1546 M^–1^ cm^–1^ at 325 nm),^10^ the spectra below 320 nm was masked by strong contributions from Trp and Tyr residues. In this regard, α_3_C clearly stands out as it is completely devoid of aromatic amino acids and rich in charged amino acids. Thus, the spectral features of α_3_C, even in the 250–300 nm range, are novel as they do not arise from aromatic chromophores. Further, the report of absorption beyond 350 nm for a monomeric protein lacking aromatic amino acids or active site chromophores is unprecedented.

The ProCharTS spectra, demonstrated above for α_3_C, is generally applicable for the study of natural proteins rich in charged amino acids irrespective of their aromatic amino acid content. In Table 1 we present new data for two proteins HSA and HEWL for which absorption spectra were previously reported up to 350 nm.^10^Table 1 shows that both α_3_C and HSA (both rich in charged amino acids) display absorption features which match well above 300 nm and extend up to 800 nm. In contrast, HEWL which has a lower percentage of charged residues in its sequence relative to HSA or α_3_C, lacks significant absorption features beyond 320 nm (Table 1). While HSA contains Tyr, Trp, and Phe residues, the absorption from these aromatic amino acids is expected to sharply drop beyond 320 nm. In contrast, the ProCharTS spectrum extends up to 800 nm providing signatures well resolved from that for Tyr and Trp in HSA. Thus it is evident that even in presence of aromatic amino acids, contribution from ProCharTS persists and remains conspicuous between 320 and 800 nm. Further, the presence of ProCharTS has implications even for the spectral ranges overlapping with that from aromatic amino acids. The significant absorption from charged amino acids around 280 nm (α_3_C shows *ε* = 4531 ± 133 M^–1^ cm^–1^ at 280 nm) should be taken into account when interpreting aromatic amino acid absorbance at 280 nm to quantify protein solutions (*e.g.* Near UV absorbance at 280 nm to estimate protein concentrations 27). Further, the ProCharTS profile broadly overlaps with the emission profile of fluorescent chromophores (such as Trp) or dyes. Thus, in addition to monitoring the absorption profile changes directly, the decay kinetics of fluorescent probes may also be used as a spectral marker to follow the dynamics and interactions of charged amino acids within protein folds.

**Table 1:** Comparison of molar extinction coefficients of α_3_C with two proteins (HSA and HEWL) for which absorption spectra was reported up to 350 nm in earlier studies. We present new data for these proteins between 350 and 800 nm. The numbers in square brackets indicate the standard deviation for *n* = 3–5

Protein (total residues)	Number and (fraction) of charged amino acids	Aromatic amino acid content	Molar extinction coefficient^*a*^ (M^–1^ cm^–1^)						
			315 nm	320 nm	350 nm	450 nm	600 nm	750 nm	800 nm
HSA (585)	197 (33.6%)	18Y; 1W; 31F	2481 [276]	2268 [232]	1460 [191]	512 [136]	333 [140]	199 [24]	198 [51]
α_3_C (67)	36 (54%)	0Y; 0W; 0F	1727 [164]	1660 [163]	1396 [158]	964 [129]	686 [98]	537 [75]	501 [67]
HEWL (129)	27 (21%)	3Y; 6W; 3F	191 [8]	148 [27]	<50^*b*^	<50^*b*^	<50^*b*^	<50^*b*^	<50^*b*^

^*a*^Measured in deionized water.

^*b*^Too low to be measured accurately.

To examine the role of the protein fold in producing the observed spectral features of α_3_C (Fig. 2a), we studied the absorption spectra of a mixture of α_3_C amino acids at proportions (see Table S2 of ESI† for amino acid concentrations) present in a 105 μM protein solution. These samples do not show any significant absorption in the 250–800 nm region in stark contrast to α_3_C polypeptide chain linking together the same amino acids (Fig. 2b). This implies that the protein fold may play a crucial role in the origin of the observed novel UV-Vis spectral features. In the next section we carry out further experimental studies to highlight the role of charged amino acids and the protein fold in producing the absorption spectrum of α_3_C in Fig. 2a.

### Charged amino acid and peptide solutions show significant absorption above 350 nm

**Fig. 3:**
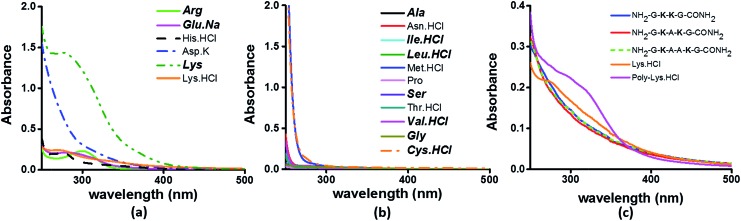
Absorption spectra of (a) charged amino acids; (b) uncharged amino acids; (c) peptides (4 mM), Lys·HCl (1 M) and poly-Lys·HCl (12.5 mg mL^–1^) in deionized water. The concentration of amino acids used was 1 M. Amino acids captioned in bold italics in (a) and (b) are constituents of α_3_C (see also Table S2†).

To examine the sequence specificity of the UV-Vis absorption from α_3_C, we studied the absorption spectra of high concentration solutions of all non-aromatic amino acids including those present in the α_3_C sequence (Fig. 3). We find significant absorption between 250 and 400 nm for charged amino acid solutions of Lys, Glu monosodium salt (Glu·Na), Arg, Asp potassium salt (Asp·K) and His (Fig. 3a). In contrast, uncharged amino acid solutions of Ala, Asn, Ile, Leu, Met, Pro, Ser, Thr, and Val show negligible absorption in this range (Fig. 3b). Note that Lys·HCl has a molar absorptivity ∼6 times smaller than that for pure Lys solutions lacking the hydrochloride ion. The decrease in absorption due to the presence of ions supports participation of the charged amino acid sidechains in the photoinduced electronic transitions. For instance, the hydrochloride ion may screen the sidechain charge to reduce the net absorption of the sample. A similar reasoning suggests that pure Glu (insoluble in aqueous medium) should have a higher molar absorptivity than that measured for its monosodium salt solution. Since Lys·HCl and Glu·Na have very similar absorption intensities (0.23 and 0.20 respectively, at 270 nm), the molar absorptivity of pure Glu may match that of pure Lys.

Previously, proton-transfer as well as the hydrogen bonding had been suggested by Pinotsi *et al.*^17^ as possible mechanisms for fluorescence of amyloid aggregates containing β-sheet architectures and lacking aromatic amino acids. However, we found no dependence of the absorption features (*ε*) of high concentration Lys solutions on the pH of the medium over a broad pH range of 2–12.5 covering the Lys amino group p*K*_a_ (Fig. S4 of ESI†). The insensitivity of the absorption features on pH argues against the participation of protons in the initial photoexcitation process of high concentration charged amino acid solutions. While Pinotsi *et al.* do not provide data on the effect of pH on the excitation spectra,^17^ our observations do not rule out the possibility of participation of hydrogen bonding or proton transfer reactions in the subsequent excited state relaxation and fluorescence processes.

The results in Fig. 3 clearly suggest a possible role for sidechains of charged amino acids (Lys, Arg, Asp, Glu, His) behind the UV-Vis absorption between 250 and 400 nm. However, there are notable differences in the spectral features in Fig. 3a *versus* the absorption profiles of the α_3_C protein (Fig. 2a and b). The tail of the charged amino acid spectra (beyond 320 nm) extends up to ∼500 nm. Short peptides containing Lys placed at different separations in the peptide sequence and poly-l-Lys·HCl solutions also show similar absorption features (Fig. 3c). In contrast, the long tail of the α_3_C absorption spectrum extends up to 800 nm. Thus, a possible role of the protein fold in the origin of tail spectra between 400 and 800 nm (Fig. 2a) is anticipated. Further, a comparison of molar extinctions coefficients of pure Lys amino acid solutions (*ε* = 1.42 M^–1^ cm^–1^ at 270 nm) with that for α_3_C (*ε* = 5808 M^–1^ cm^–1^ at 270 nm) reveals that ∼4000 fold enhancement in absorptivity at 270 nm is achieved by the folded α_3_C protein structure. We note, however, that α_3_C also contains other charged amino acids (Glu and Arg) besides Lys. To summarize, the results in this section demonstrate the ability of charged residues either in amino acid form or within extended peptide chains to absorb in the near UV. The protein fold further enhances the spectral range for the charged amino acids and the subsequent sections examine the possible mechanisms of enhancement.

### Computed UV-Vis absorption spectra for Lys and Glu monomers show charge transfer transitions

**Fig. 4:**
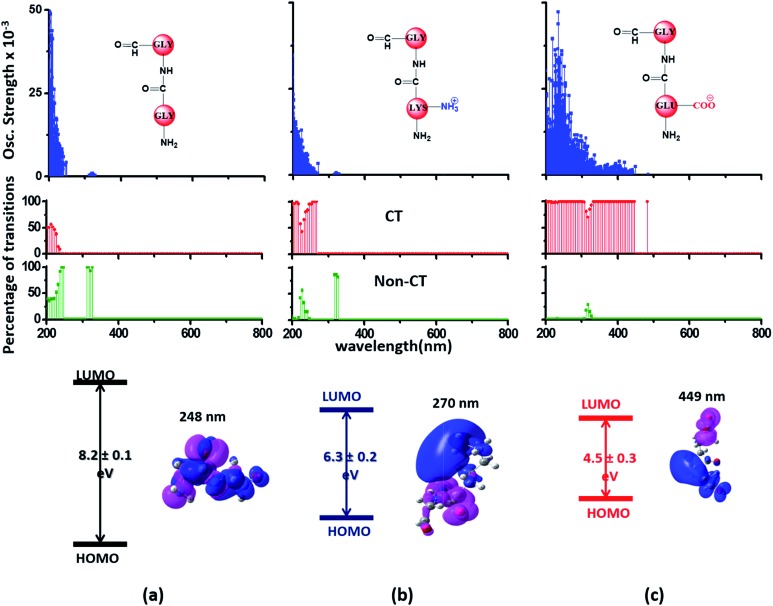
Simulated absorption spectra (wavelength *vs.* oscillator strength) for (a) Gly (control), (b) Lys and (c) Glu monomers (top row). Each panel displays spectra from 100 amino acid structures extracted from 100 ns MD trajectories of α_3_C. Each amino acid fragment (chemical structures shown) has an extended backbone capped with hydrogen atoms (see Methods). The middle row shows the assignment of transitions in the spectra to CT *vs.* non-CT transitions across the absorption profile. The bottom row summarizes the average ground state HOMO–LUMO gap (note that the lowest energy transitions are not pure HOMO → LUMO transitions; see Fig. S8 of ESI†) along with its standard deviation for the 100 snapshots used to compute each spectra. We also show difference density plots (Pink lobes represent reduction in electron density and blue lobes represent increase in electron density) for the lowest energy transitions for each spectra in the top row.

Since the α_3_C protein is rich in both Glu and Lys, we carried out TDDFT electronic structure calculations on 100 structures of each charged amino acid sampled from MD simulations of α_3_C (see Methods for a discussion on our modelling assumptions) to simulate their absorption spectra between 200 and 800 nm (Fig. 4 top row). Here we discuss the spectra of monomers with their backbone amide units capped with Gly to represent the extended backbone present in the protein environment (see Methods for our capping strategy). The application of electronic structure calculations to MD sampled structures has proven to be effective for calculating UV-Vis spectra and electronic couplings for CT in organic molecules.^39^,^40^,^48^–^50^ We visualize the lowest energy transitions of Lys, Glu, and Gly through difference density plots which show the location of hole (pink) and electron (blue) density on each amino acid fragment (Fig. 4 bottom row).

The computed Gly spectra (Fig. 4a) extends up to ∼250 nm with the lowest energy transition at 248 nm delocalized over the entire backbone unit. A decomposition of the transition into constituent single orbital transitions (Fig. S8 of ESI†) reveals that it predominantly involves the frontier orbitals (HOMO/LUMO/LUMO+5) delocalized over the backbone. Note that the weak transitions above 300 nm are spurious, arising due to the truncated form of the peptide backbone used in our calculations (see discussion below about the capping group effects). The simulated spectrum of Lys monomer (Fig. 4b) shows transitions in the same spectral range as the Gly control (Fig. 4a) extending to slightly higher wavelengths (up to 270 nm). In contrast, the Glu monomer spectra is distinct, displaying prominent transitions up to 450 nm (Fig. 4c). Difference density plots for the lowest energy Lys transitions around 270 nm show the electron density decreases (pink) on the peptide backbone and increases (blue) on the charged amino group and sidechain of Lys (Fig. 4b). This represents a CT transition involving the Lys backbone and its charged amino group. The positive charge on the Lys sidechain amino group, makes it a favorable location for the frontier unfilled orbitals of Lys (*e.g.* the LUMO in Fig. S8 of ESI†), thereby populating the amino group with charge acceptor states. The average HOMO–LUMO gap for the Lys structures is reduced by ∼2 eV with respect to that for Gly (Fig. 4 bottom row). Thus, photoinduced CT transitions should be characteristic and unique to amino acids with charged sidechains and their derivatives. Since CT transitions are highly sensitive to the nature of the charge donor/acceptor states and the chemical structure of the sidechain separating them, each charged amino acid (Lys, Glu, Arg, Asp, and His) is expected to show distinct absorption features. Indeed, the Glu spectra (Fig. 4c) shows transitions over a much greater spectral range relative to Lys, extending into the visible wavelength range (up to 450 nm). Further, since the Glu sidechain is negatively charged, the filled orbitals (*e.g.* the HOMO in Fig. S8 of ESI†) are placed on the carboxylate group. Thus, for Glu, the direction of photoinduced CT is opposite that for Lys, from the sidechain carboxylate group to the polypeptide backbone (Fig. 4c bottom row). Differences between the charged sidechain groups (carboxylate COO^–^ for Glu *vs.* amino NH_3_^+^ for Lys), different extents of hyperconjugation involving the charged groups, the presence of lone pair electrons for Glu, and the shorter sidechain for Glu (2 CH_2_ links *vs.* 4 in Lys), all contribute towards the difference in spectral features for Lys and Glu monomers. The average HOMO–LUMO gap for the Glu structures is reduced by ∼4 eV and ∼2 eV with respect to that for Gly and Lys respectively.

We characterized all transitions in the simulated spectra for each amino acid through two measures of spatial separation of charges (see Methods): (1) charge separation indices (Δ*r*)^36^ given by eqn (1), and (2) distance between hole and electron centroids (*D*_CT_)^34^,^37^ given by eqn (2). The middle rows in Fig. 4 show the percentage of CT *vs.* non-CT transitions within 5 nm wavelength windows over the whole absorption spectral range for all three amino acids. The data show that transitions above 200 nm for Glu and Lys monomers are rich in CT transitions. It is of course possible to get significant photoinduced charge separation on the extended polypeptide backbone, so that CT transitions are not exclusive to charged amino acids. For instance, the control Gly spectra (Fig. 4a) also seem to produce CT transitions above 200 nm. However, analyses of these transitions reveal that these states are actually localized backbone transitions showing spurious charge separation due to small orbital contributions of the capping groups (Fig. S7 of ESI†). However, this contamination appears to be restricted to transitions close to 200 nm and the lowest energy transitions around 250 nm are not significantly affected (see comparison of transitions around 250 nm for Gly dimer and tetramer in Fig. S5 of ESI†). Control calculations on Gly tetramers and Gly dipeptides truncated with methyl capping show (Fig. S6 of ESI†) that the spurious CT character of the transitions above 200 nm are diminished along with a blue shift in the spectra. In our calculations on tetramers and dipeptides, we find CT transitions around the 150–180 nm wavelength range (Fig. S6 of ESI†) consistent with previous computational reports of dipeptide and tripeptide spectra.^32^,^38^,^51^ Finally, a small number of spurious non-CT transitions which are completely localized on capping groups (Fig. 4 and S7 of ESI†) appear consistently around 320 nm in the spectra of all three amino acids (Gly, Lys and Glu) which can be clearly identified and distinguished from CT transitions. To assess the impact of the capping groups on the signature CT transitions of charged amino acids, we carried out control calculations on Glu peptides with different backbone extensions/capping (Fig. S6 of ESI†). These control calculations show that the effect of the capping group is negligible for transitions beyond 250 nm. For the Glu spectra, the most pronounced changes with change in backbone extension occur around 200 nm for transitions localized on the backbone (consistent with results for Gly peptides in Fig. S6 of ESI†). In contrast, the prominent CT absorption of Glu at lower energies (above 250 nm) is not altered in terms of both peak intensities and the spectral range (Fig. S6 of ESI†). We thus conclude that the effect of the capping groups is mostly restricted to backbone transitions around 200 nm with negligible effect on the signature backbone/sidechain CT transitions of the charged amino acids above 250 nm.

To summarize, charged amino acids, Lys and Glu, in monomeric form produce characteristic CT transitions. The intensities and spectral range of transitions for monomeric Lys and Gly with extended backbone are very similar and these amino acids are not distinguishable on the basis of their absorption spectra. The electronic properties of the monomeric Lys/Glu chromophores is neither able to explain the full spectral range of the transitions seen in high concentration Lys solutions (250–400 nm) nor that seen for the α_3_C protein (extending up to 800 nm). In the following sections, we explore higher order sidechain interactions between the charged amino acids within α_3_C which shed light on the role of the protein fold in dramatically extending the spectral range of Lys/Glu CT transitions.

### MD simulations of α_3_C reveal significant interactions between Lys and Glu sidechains

**Fig. 5:**
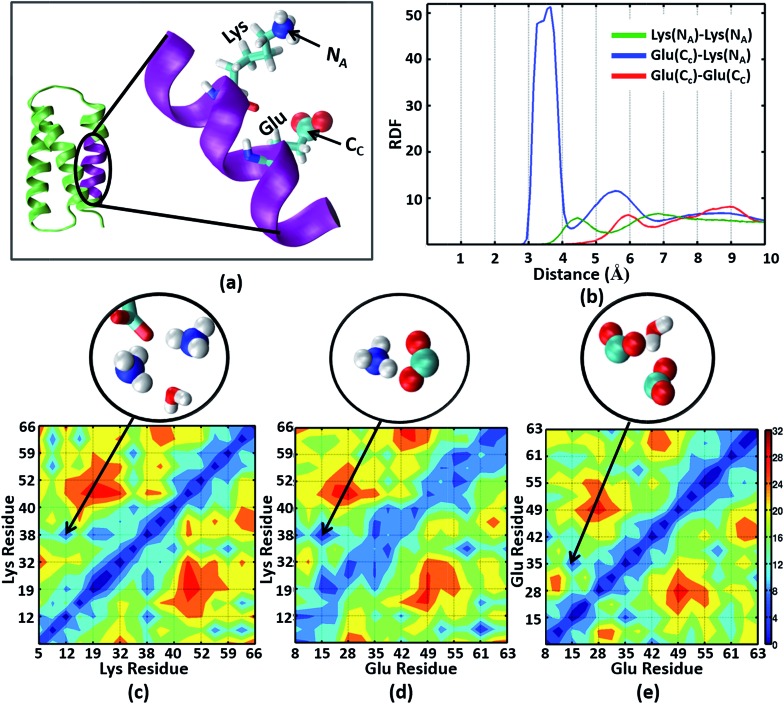
(a) The α_3_C protein (green) and an enlarged view of one of its helical segments (purple) containing a Lys and Glu residue. The Lys amino nitrogen N_A_ and Glu carboxylate carbon C_C_ atoms are marked. (b) Radial Distribution Function (RDF) plots for N_A_–N_A_, C_C_–N_A_, and C_C_–C_C_ atom pairs. The lower panels show contact maps (pairwise distance averages over the MD trajectory) for (c) N_A_–N_A_, (d) C_C_–N_A_, and (e) C_C_–C_C_ atom pairs. Representative interactions for the region marked in the contact maps are explicitly shown in the circled images extracted from MD snapshots.

The NMR structures for the α_3_C protein (Fig. 1a) show several Lys and Glu residue pairs placed in close proximity. We thus investigated the interactions of Lys/Glu sidechains within the α_3_C protein fold using classical atomistic MD simulations of the solvated protein (see Methods). As discussed previously, even at the maximum concentration of α_3_C employed in our experiments (105 μM), we expect the protein to remain in monomer form. Accordingly, our simulations comprised of a single α_3_C molecule immersed in water box of volume ∼22 500 Å^3^ with periodic boundary conditions. We generated radial distribution function (RDF) plots capturing the range of pairwise atomic separations in our MD simulation trajectory (see Fig. 5a): (1) Lys amino nitrogen (N_A_–N_A_) atom pairs, (2) Glu carboxylate carbon and Lys amino nitrogen (C_C_–N_A_) atom pairs, and (3) Glu carboxylate carbon (C_C_–C_C_) atom pairs. Further, we created 2-D contact maps displaying the average separations for these atom pairs representing Lys–Lys, Glu–Lys, and Glu–Glu sidechain interactions over the MD trajectory.

The Lys N_A_–N_A_ RDF plot (Fig. 5b) shows peaks around 4.5 Å and 7 Å which is surprising as two positively charged sidechains should repel each other. This observation is reinforced in the Lys–Lys contact map (Fig. 5c) which reveals multiple sets of amino group interactions, wherein the average N_A_–N_A_ separation is lower than 7 Å over the MD trajectory. A visualization of the dynamics of Lys residue pairs during the MD trajectory reveals that the interactions of Lys amino groups are mediated either by water molecules (Movie S9 in ESI†), or by Glu residues (Movie S10 in ESI†), or by both (Fig. 5c circled image). Glu residues can indirectly mediate Lys–Lys sidechain interactions by screening the Lys charge. Water molecules mediate Lys–Lys sidechain interactions through hydrogen bonding and by screening the Lys amino group charges as hypothesized previously.^10^ We note that the mediation of charged sidechain interactions by polar and charged chemical species (water, Glu sidechains) will also include electronic effects which are not captured by MD simulations^52^ but can significantly modulate spectral features. We discuss such effects in the subsequent sections. The C_C_–N_A_ RDF shows a peak around 3.5 Å (Fig. 5b), corresponding to strong salt bridge interactions between the Lys amino group and the Glu carboxylate group. The time scales associated with Lys–Lys or Glu–Lys sidechain interactions vary from picoseconds to a few nanoseconds in our MD trajectory (Fig. S11 of ESI†). The Glu C_C_–C_C_ RDF plot shows peaks at ∼6 Å and ∼9 Å, indicating weaker interactions between Glu sidechains relative to that between Lys sidechains. Note that sidechain interactions for α_3_C include both nearest neighbor residue pairs (NN pairs) or distally separated residue pairs (DS) in the protein sequence (Fig. 1b). Both DS and NN interactions tend to show similar separations between the amino/carboxylate groups (Fig. S12 in ESI†), but the electronic coupling strengths for such interactions differ so as to produce significant spectral differences (*vide infra*).

### Interactions between Lys and Glu sidechains can extend the spectral range of CT transitions from charged amino acids to wavelengths above 300 nm

**Fig. 6:**
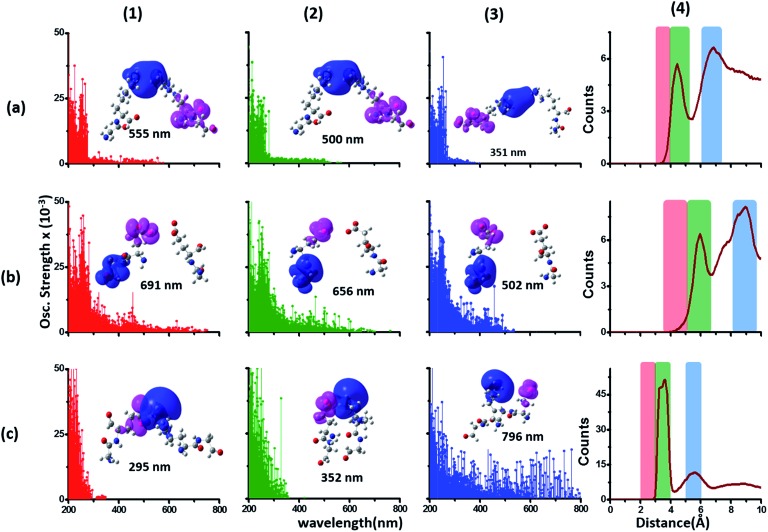
Simulated absorption spectra (wavelength *vs.* oscillator strength) for distally separated in sequence (DS) dimers (a1–a3) Lys–Lys, (b1–b3) Glu–Glu, and (c1–c3) Lys–Glu. Each panel displays spectra from 100 amino acid structures extracted from 100 ns MD trajectories of α_3_C. Each amino acid fragment in the dimer has an extended backbone capped with hydrogen atoms (see Methods). Row (a) Lys–Lys pair with (1) 3–4 Å (red), (2) 4–5.5 Å (green) and (3) 6–7.5 Å (blue) separations of Lys amino groups chosen from shaded regions of the radial distribution function (RDF) plot (4) of Lys N_A_ atom pairs. Row (b) Glu–Glu pair with (1) 3.5–5 Å (red), (2) 5–6.5 Å (green) and (3) 8–9.5 Å (blue) separations of Glu carboxylate groups chosen from shaded regions of the RDF plot (4) of Glu C_C_ atom pairs. Row (c) Lys–Glu pair with (1) 2–3 Å (red), (2) 3–4 Å (green) and (3) 5–6 Å (blue) separations of Lys amino and Glu carboxylate groups chosen from shaded regions of the RDF plot (4) of Lys N_A_ and Glu C_C_ atom pairs. In each absorption spectra panel (a1–a3, b1–b3 and c1–c3) difference density plots show regions with decrease in electron density (pink lobes) and regions with increase in electron density (blue lobes) on Lys/Glu fragments for the lowest energy transitions in that panel.

We generated TDDFT based 200–800 nm spectra (see Methods) for NN and DS Lys–Lys, Glu–Glu and Lys–Glu residue pairs sampled from the α_3_C MD trajectory. In these calculations we retained an extended dimer backbone for DS fragments (details in Methods) wherein the backbone of each of the two residues of a DS pair was extended to include the backbone of the adjacent peptide units. We examined data (Fig. 6) for DS pairs (corresponding data for NN pairs are shown in Fig. S13 of ESI†) for three different separations of amino/carboxylate groups chosen on the basis of RDF data (Fig. 6). Specifically, for each Lys–Lys, Glu–Glu, and Glu–Lys residue pair, we processed 100 conformations each with N_A_–N_A_, C_C_–C_C_, and C_C_–N_A_ atom pair separations corresponding to red (strong interactions), green (intermediate interactions) and blue (weak interactions) shaded ranges in the corresponding RDF plots (Fig. 6 panels (a4–c4)). Both Lys–Lys and Glu–Glu sidechain interactions create new low energy transitions (panels (a1–a3) and (b1–b3) in Fig. 6) which extend the absorption range seen for Lys and Glu monomers by 100–300 nm towards the visible region. The spectral range for DS Lys–Lys pairs (Fig. 6, panels (a1–a3)) extends up to 550 nm (strong interactions), 500 nm (intermediate interactions), and 350 nm (weak interactions). The NN data (Fig. S13 of ESI†) show similar but weaker extensions of the spectral range for interacting Lys–Lys pairs; corresponding spectral ranges for NN interactions (Fig. S13 of ESI†) are curtailed to 450 nm (strong interactions), 440 nm (intermediate interactions), and 310 nm (weak interactions), respectively. Similar trends are observed for DS *vs.* NN Glu–Glu pair spectra (Fig. 6 panels (b1–b3) and S13 of ESI†). Characterization of transitions in the spectra on the basis of charge separation measures (eqn (1) and (2)) for Lys–Lys and Glu–Glu interacting pairs (both DS and NN) reveals mostly CT transitions beyond 250 nm (Fig. S15 of ESI†). For both DS and NN pairs, the lowest energy transitions involve CT between the extended backbone and the amino/carboxylate group of Lys/Glu (difference density plots in Fig. 6 panels (a1–a3) and (b1–b3), S13 of ESI†). A decomposition of the lowest energy transition for the most strongly interacting DS Lys–Lys pairs shows that (Fig. S14 of ESI†) these transitions are predominantly frontier orbital transitions with the LUMO delocalized between the interacting amino groups. For DS and NN Glu–Glu pairs, the direction of CT is opposite to that seen for Lys–Lys pairs with the charge donor states (pink) located on sidechain carboxylate groups. For both NN and DS Glu–Glu pairs, interactions between sidechain carboxylate groups are weaker than that for Lys amino groups. A decomposition of the lowest energy transitions (Fig. S14 of ESI†) for the most strongly interacting Glu–Glu pairs reveals that while these transitions are predominantly frontier orbital transitions, the HOMO is localized to one of the carboxylates of the interacting Glu–Glu pairs.

The interactions between Lys–Glu residue pairs lead to starkly different spectral profiles from that produced by Lys–Lys and Glu–Glu interactions. Weakly interacting Lys–Glu residues produce prominent transitions up to 800 nm, whereas strong interactions between the oppositely charged amino and carboxylate groups limit the transitions to 350 nm in the computed spectrum (Fig. 6 panels (c1–c3)). For weakly interacting Lys–Glu interactions, the lowest energy transitions involve CT transitions from the negatively charged (charge donor) Glu carboxylate group to the positively charged (charge acceptor) Lys amino group (Fig. 6 panel (c3) and S13 of ESI†). In contrast, strong interactions between Lys amino and Glu carboxylate groups create a neutral moiety through the formation of a salt bridge which curtails the spectral range of CT transitions (Fig. 6 panels (c1) and (c2)). Thus, in contrast to the Lys–Lys/Glu–Glu case, extension of CT spectral range to lower energies is inversely proportional to the strength of DS Lys–Glu interactions. We find that the NN Lys–Glu pairs are unable to form salt bridges due to geometry constraints and therefore show only weak interactions with spectral features that extend beyond 800 nm in the computed spectrum (Fig. S13 of ESI†).

To summarize, the results in this section show that the association of charged sidechain amino/carboxylate groups in Lys/Glu residue pairs can greatly extend the spectral range of CT transitions observed for Lys/Glu monomers beyond 300 nm.

### Mechanism of CT transitions in α_3_C and modulation of absorption features by the protein and solvent environment

**Fig. 7:**
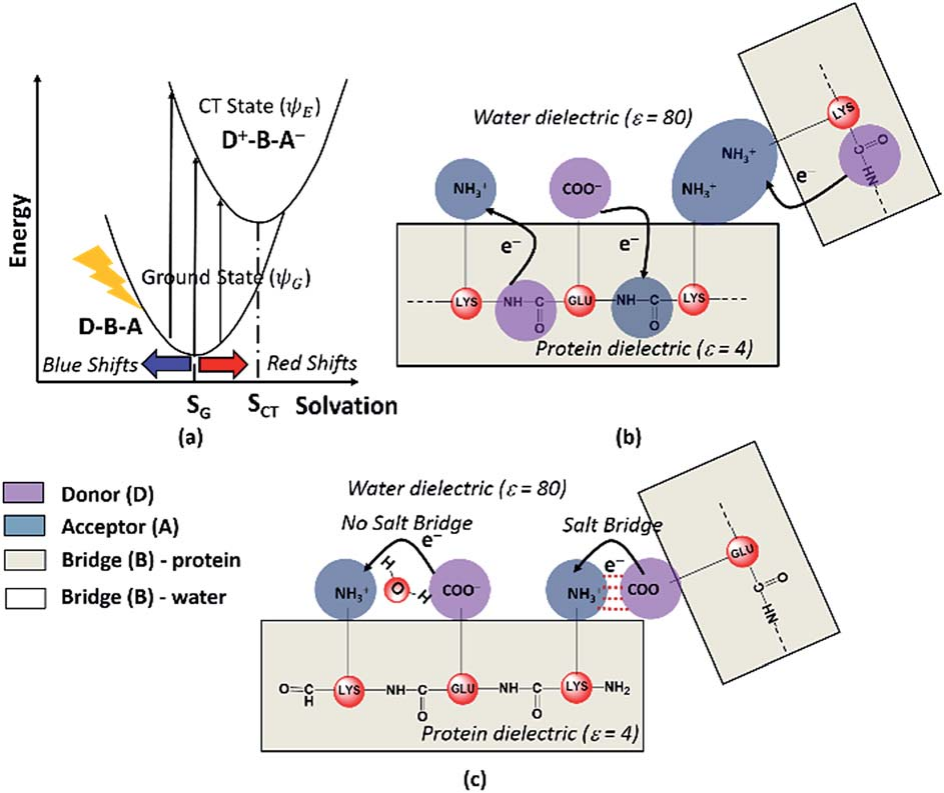
(a) Dependence of photoinduced CT transition energies for a general Donor (D)–Bridge (B)–Acceptor (A) system plotted with respect to a general solvation coordinate. Minimum energy solvent configurations for the ground and excited states are referred to as *S*_G_ and *S*_CT_ respectively. Schematic describing (b) Peptide Backbone–Sidechain (PBS) and (c) Sidechain–Sidechain (SS) CT transitions captured in the present study. Close interactions between sidechains with like charges (amino groups in panel (b)) lead to more delocalized A states and lower the CT transition energy. In contrast close interactions between sidechains with unlike charges (amino-carboxylate salt bridges in panel (c)) can shift the photoinduced CT transitions to higher energies.

Photoinduced CT can be described in the framework of a three component Donor(D) – Bridge(B) – Acceptor(A) molecular complex^50^,^53^,^54^ wherein the D and A components are electron donating and electron accepting groups respectively. In contrast, the B component electronically couples the D and A components. The absorption of light by such a molecular complex can lead to a CT transition when electrons are transferred from D to A. The photoinduced CT may either proceed through the creation of a locally excited state on the donor or directly transfer charge from the donor to the acceptor. The CT transitions in charged amino acids subscribe to the latter model (Fig. 7a). While solvation coordinates couple to both donor and acceptor states, the vertical CT transition energies depend only on the ground state solvation configuration (Fig. 7a). The electronic coupling between D and A which determines the relative energies of the ground (*ψ*_G_) and excited (*ψ*_E_) state depends critically on the chemical structure of B. Thus, a quantitative description of intensities and peak positions of CT transitions should include a rigorous description of both solvation and the D–A electronic coupling through B states.

In our study, we propose two different types of CT transitions (Fig. 7) to explain the α_3_C absorption profile: (1) peptide backbone to sidechain CT (PBS-CT), and (2) sidechain to sidechain CT (SS-CT). In PBS-CT, the peptide backbone and the charged amino/carboxylate groups of Lys/Glu residues act as the D/A components, and the sidechain alkyl groups act as the B component of the D–B–A complex (Fig. 7b). In SS-CT, the negatively charged Glu carboxylate groups act as D and the positively charged Lys amino groups act as A (Fig. 7c). Here, however, the B component is variable and depends on protein and solvent dynamics. Depending on the D–A distance, the B component could include variable number of water molecules and/or other sidechain groups. For both (PBS-CT and SS-CT) transitions the Lys/Glu charge plays a crucial role in dictating the direction of charge transfer. In the previous sections, we showed that Lys/Glu CT transition energies may be shifted to lower energies (above 300 nm) due to pairwise interactions with NN or DS Lys/Glu residues. The polar protein environment can further influence the CT energies of such charged amino acid dimers by introducing higher order interactions involving other charged sidechains and/or bound water. Further, the p*K*_a_ of some of the interacting sidechains may be altered. Below we first discuss how the dimer interactions lead to the spectral shifts observed for charged amino acids and then discuss the higher order effect of the environment on the spectra.

The photoinduced CT in monomer Lys and Glu residues is a PBS-CT process (Fig. 7b). Association of charged sidechains can modulate PBS-CT (DS Lys–Lys example in Fig. 7b) by altering the relative stabilities of the *ψ*_G_ and *ψ*_E_ as a function of distance between these groups. The association of groups with like charges (Lys–Lys amino groups or Glu–Glu carboxylate groups) should destabilize *ψ*_G_ due to unfavorable electrostatics. In contrast, such associations should stabilize *ψ*_E_ due to a higher probability of placing/removing electrons from the Lys amino/Glu carboxylate groups during PBS-CT. The net result is a lowering of the energy gap for photoinduced PBS-CT transitions, commensurate with decreasing distance between charged sidechains. The average HOMO–LUMO gap for Lys–Lys dimers (Fig. S16 of ESI†) is lowered by ∼1 eV as the distance between their amino groups is reduced from around 6–7 Å to 3–4 Å. Likewise, the average HOMO–LUMO gap for Glu–Glu dimers (Fig. S16 of ESI†) is reduced by ∼0.7 eV as their carboxylate group separation reduces from around 8–10 Å to 4–5 Å. In contrast, when groups with unlike charges interact, the mechanism of CT changes to SS-CT (Fig. 7c). In this case strong interactions (Lys–Glu salt bridges) should stabilize *ψ*_G_ (favorable electrostatics) and destabilize *ψ*_E_ (neutralization of charges). Thus, for Lys–Glu interactions the SS-CT transition energy is lowered commensurate with increasing distance between the amino acid sidechains. The average HOMO–LUMO gap for Lys–Glu dimers (Fig. S16 of ESI†) increases by more than 2.5 eV as the Lys–Glu amino-carboxylate distance reduces from around 5–6 Å to the salt-bridge forming distance (3–4 Å).

**Fig. 8:**
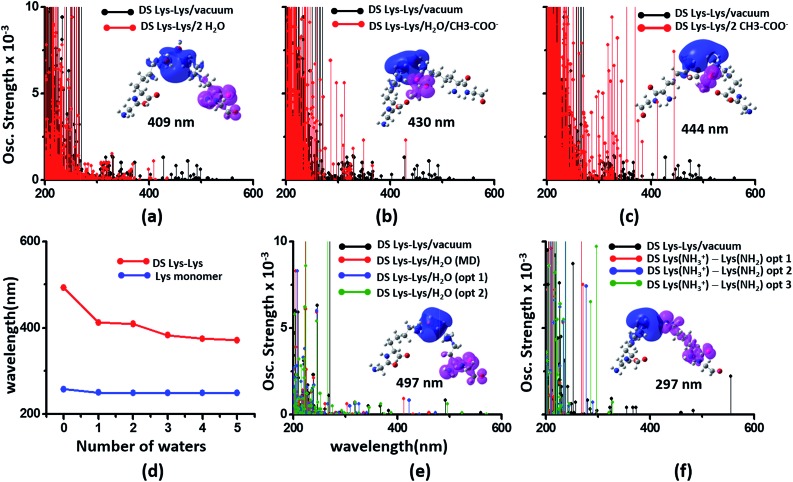
Modulation of DS Lys–Lys dimer absorption due to the environment (corresponding data for Glu–Glu and Lys–Glu in Fig. S17 and S18 of ESI†): (a) vacuum *vs.* 2 waters, (b) vacuum *vs.* water + Glu carboxylate groups, (c) vacuum *vs.* 2 Glu carboxylate groups, (d) shifts in wavelengths of the lowest energy transitions with number of explicit waters included in the TDDFT calculations for monomer and dimer Lys spectra, (e) influence of water position (single explicit water) on the computed DS Lys–Lys spectral range, (f) spectra of DS Lys–Lys dimers with one of amino groups deprotonated. In panels (a–c) spectra from 10 representative conformations sampled from MD are plotted for each calculation. In panel (d) we plot the wavelength of the lowest energy transitions for DS Lys–Lys dimers and Lys monomers from TDDFT calculations on chromophores (Lys + water) from 10 representative MD snapshots. In panels (e) and (f), we show spectra calculations on a single representative DS Lys–Lys snapshot sampled from our MD simulations. In panel (e) the water was moved closer to the amino groups relative to the position sampled in MD (O–N_A_ distances of 3.09 Å, and 2.77 Å) with data for two positions shown: opt 1 (O–N_A_ distances of 2.52 Å, and 2.51 Å), and opt 2 (O–N_A_ distances of 2.15 Å, and 2.11 Å). In panel (f) we compare spectral changes after removing a single hydrogen from one of the amino groups in an interacting DS Lys–Lys pair. The three data sets opt 1, opt 2, and opt 3 correspond to removal of different hydrogen atoms in the amino group.

We next examined spectral features of charged amino acid dimer pairs in the presence of explicit water and other chemical species in the vicinity. DS Lys–Lys dimer spectra are shifted to higher energies by around 100–150 nm upon including neighboring waters and/or Glu carboxylate groups in the calculations (Fig. 8 panels (a–c)). Similar blue shifts are also seen for DS Glu–Glu and DS Lys–Glu pairs with explicit water (Fig. S17 of ESI†). While inclusion of explicit waters induces only blue shifts in the DS Lys–Lys spectra, inclusion of carboxylate groups additionally leads to more intense transitions above 300 nm. These high intensity transitions arise from photoinduced SS-CT (Fig. 8 panels (b) and (c)) between carboxylate and amino groups not present in the vacuum Lys–Lys dimer spectra. For calculations with explicit waters, the DS Lys–Lys/DS Glu–Glu spectral shifts converge upon including 5 closest waters (Fig. 8d and S17 of ESI†), retaining a red shift of ∼100/150 nm for the lowest energy transitions relative to that for Lys/Glu monomer. The difference density plots (Fig. 8 and S17†) show that nature of the lowest energy transitions are also unaltered (PBS-CT for Lys–Lys or Glu–Glu dimers and SS-CT for Lys–Glu dimers) upon inclusion of explicit water in the calculations. The spectral shifts for dimer pairs are highly sensitive to the position of waters with respect to the Lys amino groups. In Fig. 8e, we show that for the case of a single explicit water bridging the Lys–Lys pair the extent of spectral blue shifts introduced by the water can be reduced dramatically as the water is placed closer to the Lys amino groups. Similar trends are seen for Glu–Glu and Lys–Glu dimer spectra computed with explicit water (Fig. S17 of ESI†). These results clearly demonstrate that waters can both enhance and reduce the electronic coupling between charged sidechains (bridge effect *vs.* the polarization effect). We note, that a previous study comparing solvation of amino and carboxylate groups in *ab initio* and classical MD simulations showed that classical MD overestimates the number of water molecules interacting with charged groups and underestimates the electronic coupling between the charged sidechain moieties.^52^ Thus, QM/MM calculations of solvated dimers replacing waters with point charges will overestimate solvent polarization effects while ignoring bridge electronic coupling contributions. Further, our QM calculations of solvated dimers with waters sampled from classical simulations also likely overestimate solvent polarization effects while underestimating bridge electronic coupling contributions.

In our classical MD simulations of α_3_C carried out at pH 7, we assumed standard p*K*_a_ values for the Lys amino and Glu carboxylate groups. Thus, all Lys and Glu residues are charged. However, given the high concentration of charged species and their dynamic encounters, it is possible that sidechains may exchange protons to change their charged states. Thus, in addition to the dimers pairs with both sidechains charged (doubly charged pairs) it may also be possible to find Lys–Lys, Glu–Glu, and Lys–Glu dimer pairs, wherein one of the monomer sidechains is uncharged (singly charged pairs). Following the analysis of dimer spectra presented earlier in this subsection, if one of the amino acids in a Lys–Lys or Glu–Glu dimer is neutralized, we anticipate stabilization of *ψ*_G_ (less electrostatic repulsion) and destabilization of *ψ*_E_ (lower charge on D and A) leading to a spectral blue shift towards that of the charged monomer amino acid. Indeed, we find (Fig. 8f) that the DS Lys–Lys spectral range, extending up to 550 nm for doubly charged pairs, is blue shifted, extending up to ∼300 nm when one of the amino groups is deprotonated in our calculations. The lowest energy transitions are sensitive to the position of the proton shared by singly charged Lys–Lys pairs and appear much more intense (relative to doubly charged dimer spectra) between 250 and 300 nm. The difference density plots for the lowest energy transition for singly charged Lys–Lys pairs reveal that both PBS-CT and SS-CT are operational. SS-CT occurs from the uncharged to the charged Lys amino group due to the short distance between the sidechains. For Lys–Glu salt bridge pairs, deprotonation of either Lys amino groups or protonation of Glu carboxylate groups also shifts the spectra to resemble that for the charged monomer (Fig. S18 of ESI†). Thus, for short Lys–Glu separations (salt bridge), the spectra should blue shift when the carboxylate group is protonated and red shift when the amino group is deprotonated. For all singly charged dimer pairs (Lys–Lys, Glu–Glu, and Lys–Glu) with well separated sidechains, we anticipate PBS-CT within the charged monomer of the pair to be more competitive than SSCT between monomers producing spectra which resembles that for the charged monomer in the pair (see data for Lys–Glu in Fig. S18 of ESI†).

To summarize, the α_3_C spectrum (Fig. 2) can be rationalized in terms of the light absorption by a range of D–B–A chromophores involving charged amino acids and two distinct types of photoinduced CT transitions (PBS-CT and SS-CT). The chromophores show diversity in terms of the electronic character of the D, B, and A groups. We have further computed absorption profiles (Fig. S19 of ESI†) for NN Lys–AAA dimer pairs (AAA = Ala, Val, Ile, Cys and Leu), which together with the Lys–Lys, Lys–Glu, and Glu–Glu pairs represent all Lys containing NN dimer species present in α_3_C. Other than the charged amino acid dimers (Lys–Lys, Lys–Glu, and Glu–Glu) studied in this section, no other dimer species shows significant absorption beyond 300 nm. Thus, our calculations highlight the role of the association between charged amino acid side-chains in producing the long tail absorption of α_3_C above 300 nm (Fig. 2a).

### Sensitivity of UV-Vis absorption profile of α_3_C to temperature and pH induced structural changes

**Fig. 9:**
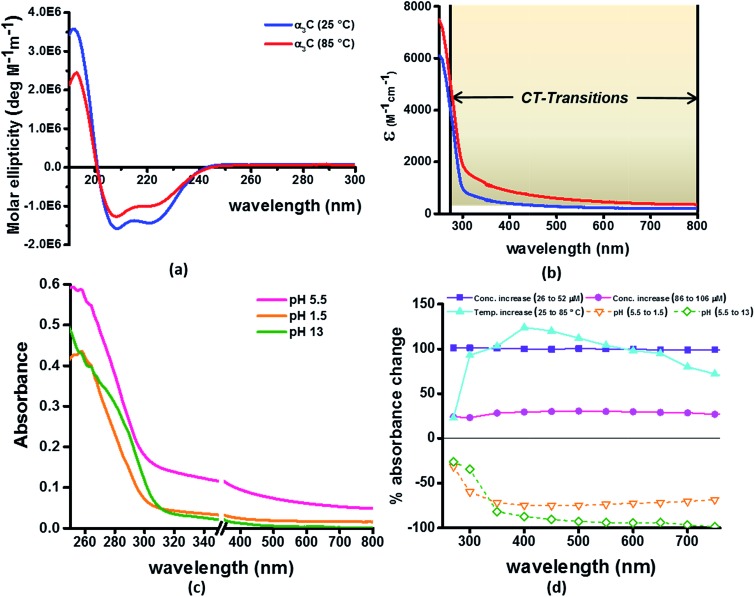
(a) CD spectra α_3_C at 25 °C and 85 °C; (b) comparison of molar extinction coefficient of α_3_C at 25 °C and 85 °C; (c) absorption spectra of α_3_C (85 μM) at different pH. An expanded scale is specifically shown to highlight changes at short wavelengths. Scale has a break between 350 and 351 nm; (d) percent change in absorbance of α_3_C at different wavelengths with increase in temperature (25 °C to 85 °C) compared with increase in concentration (26 μM to 52 μM and 85 to 105 μM) and change in pH (5.5 to 1.5 and 5.5 to 13).

Our computational results predict that the spectral range of the α_3_C ProCharTS profile should be sensitive to the interactions between Lys/Glu sidechains. Our analysis suggests two clear reaction coordinates that modulate the spectral range of ProCharTS: (1) distance between sidechains of charged amino acids, and (2) the sign of charge between interacting sidechains. Thus, we anticipate that the spectral range of ProCharTS will extend to lower energies (longer wavelengths above 300 nm) as the order of interactions between sidechains with like charges (Lys^*n*^; Glu^*n*^; *n* = order) increases. In contrast, the spectra will be curtailed to higher energies (shorter wavelengths below 300 nm) when sidechains with unlike charges interact strongly (Lys–Glu salt bridges). Based on these observations we reasoned that perturbations of the protein tertiary fold which alter the Lys/Glu sidechain interactions should modify the UV-Vis absorption spectral profile of α_3_C. To verify this, we employed two approaches. In the first approach, CD and absorption spectra for α_3_C was recorded over a temperature range of 25–85 °C. The CD spectra (Fig. 9a) reveal that the protein retains a significant fraction of its α-helical structure even at temperatures as high as 85 °C. In contrast, the UV-Vis absorption profile of α_3_C shows sensitivity to temperature (Fig. 9b) increasing by 1.2–2 fold between 300 and 500 nm. Fig. 9d shows that the temperature induced changes in spectral profile are non-uniform between 250 and 500 nm, distinct from the uniform and linear (Fig. 2a) changes induced by varying protein concentrations. The Bjerrum length (
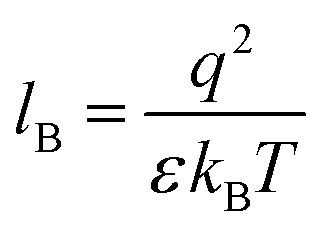
; *ε* = dielectric of the medium, *T* = temperature, *k*_B_ = Boltzmann's constant) for sidechain interactions in Lys–Lys and Glu–Glu dimers should decrease with increasing temperature as thermal energy compensates for electrostatic repulsion. In contrast, strong Lys–Glu interactions (salt bridges) should be destabilized as entropic contributions increase with temperature. Both factors, increase in Lys–Lys/Glu–Glu dimer associations and increase in Lys–Glu separations, rationalize the 90–120% increase in intensity for the α_3_C ProCharTS band between 300 and 500 nm as the temperature increases from 25–85 °C. Contributions from Lys–Lys/Glu–Glu at higher energies (200–300 nm) should also go up. However, contributions of monomers and Lys–Glu salt bridge species will decrease (increase in Lys–Lys/Glu–Glu dimer formation and increase in Lys–Glu separation) in this wavelength range as temperature increases. These compensating factors can rationalize the modest 20% increase for the spectrum around 270 nm.

In the next approach, our objective was to ascertain the role of charge in NH_3_^+^ and COO^–^ groups on the protein CT spectra. For this purpose, we altered the pH of the medium to extreme limits (pH 1 and 13), so that the protein contained only one charged species. Under these conditions, both Lys–Glu salt bridges and long range SS-CT between Lys–Glu pairs (which dominate the spectra at longer wavelengths) should not exist. Further, in absence of electrostatic attraction between oppositely charged pairs of NH_3_^+^ and COO^–^ groups, the protein structure is expected to be destabilized and likely unfolded, such that DS dimer interactions (Lys–Lys at pH 1.5 and Glu–Glu at pH 13) are reduced while NN dimer interactions (there are multiple adjacent pairs and triplets of Glu and Lys in the sequence), may still persist. NN dimer spectra (Lys–Lys and Glu–Glu only) are blue shifted with respect to that for their DS dimer counterparts (Fig. 6 and S13 of ESI†). Further, calculations on singly charged dimer pairs show spectra which are significantly blue-shifted with respect to that for their doubly charged counterparts (Fig. S18 of ESI†). Taken together, all these factors suggest that the ProCharTS absorption should be reduced at lower energies under extreme pH conditions. Indeed, Fig. 9c shows that absorption in the range 310–800 nm has nearly diminished (a dramatic >70% dip), both at pH 1.5 and 13, in comparison to the spectrum at pH 5.5.

In summary, the experimental pH variations clearly validate the critical role played by charged Lys–Lys, Glu–Glu, and Lys–Glu interactions contributed by the protein fold to the α_3_C ProCharTS absorption in the near UV-Visible range. The temperature variation, on the other hand, emphasizes the sensitivity of the α_3_C ProCharTS absorption intensity to perturbation of tertiary Lys–Lys, Glu–Glu and Lys–Glu sidechain contacts. In order to extract detailed structural information on proteins from ProCharTS, a careful computational mapping of spectral intensities and peaks to geometric parameters (distances and angles) of specific chromophores is required. Such mappings should account for spectral shifts due to the environment (see previous subsection) and must be benchmarked against experimental constraints. Our study represents a first step in this direction and opens the door for both computational and experimental investigations for mapping the ProCharTS spectral profile to biomolecular structure and dynamics.

## Conclusions

Using α_3_C as a model, we have unambiguously demonstrated that monomeric proteins lacking aromatic amino acids can display significant UV-Vis absorption with notable features between 250 and 300 nm and a long tail that can extend up to 800 nm (Fig. 2). We have presented several lines of evidence (both experimental and theoretical) to show that charged amino acids (Lys and Glu) can produce the observed spectral features. Through experimental control studies on high concentration solutions of non-aromatic amino acids and Lys containing peptides (Fig. 3), we showed that charged amino acids possess distinctive spectral features beyond 250 nm. Our computational analysis on Lys and Glu amino acids extracted from MD generated structures of α_3_C revealed CT transitions between 250 and 450 nm in the computed TDDFT spectra (Fig. 4). The CT transitions involve the amino (NH_3_^+^)/carboxylate (COO^–^) groups of Lys/Glu sidechains and the peptide backbone. Classical MD simulations revealed dimer and higher order interactions between Lys amino and Glu carboxylate groups imposed by the protein fold (Fig. 5). The interactions between charged amino acid sidechains were found to strongly modulate the computed CT absorption spectral profile (Fig. 6) and can account for the broad 250–800 nm absorption of α_3_C (Fig. 2). We described two specific mechanisms of photoinduced CT (PBS-CT and SS-CT) involving Lys and Glu amino acids which are operational in α_3_C (Fig. 7) and their modulation by the polar solvent/protein environment (Fig. 8). Finally, we experimentally demonstrated the sensitivity of the α_3_C absorption spectrum to temperature and pH induced structural changes of the protein fold (Fig. 9). Our results connect UV-Vis absorption in proteins to the charged amino acid content of protein sequences for the first time and rationalize hitherto unexplained experimental observations of absorption beyond 300 nm in Lys-rich proteins. The novel assignment of CT transitions to the 250–800 nm region in the absorption profile of proteins opens up a new spectral window (ProCharTS) to develop intrinsic spectral markers to monitor structure and dynamics of proteins rich in charged amino acids, such as nucleic acid binding proteins or intrinsically disordered proteins, irrespective of their aromatic amino acid content.

## Supplementary Material

Supplementary information

Supplementary movie

Supplementary movie
