# Supplementary material for: Near UV-Visible electronic absorption originating from charged amino acids in a monomeric protein
Source: Chem Sci. 2017 May 19;8(8):5416–33. doi: 10.1039/c7sc00880e (PMC5609530; doi:10.1039/c7sc00880e)
Supplement: Supplementary file 1 [file SC-008-C7SC00880E-s001.pdf]

Electronic Supplementary Material (ESI) for Chemical Science.
This journal is © The Royal Society of Chemistry 2017

Supporting Information

Near UV-Visible Electronic Absorption Originating from Charged Amino Acids in a Monomeric Protein

Saumya Prasad[†#], *Imon Mandal*[‡#], *Shubham Singh*[†], *Ashim Paul*[⊥], *Bhubaneswar Mandal*[⊥], *Ravindra Venkatramani*[‡]*, *Rajaram Swaminathan*[†]*

†Department of Biosciences and Bioengineering, Indian Institute of Technology Guwahati, Guwahati 781039, Assam, India

‡Department of Chemical Sciences, Tata Institute of Fundamental Research, Homi Bhabha Road, Colaba, Mumbai 400 005, India.

⊥Department of Chemistry, Indian Institute of Technology Guwahati, Guwahati 781039, Assam, India

# These authors contributed equally

* Corresponding Authors

CONTENTS

	Page Number
1. Details of Experimental and Computational Methods	2-10
2. Amino acid concentrations in 105 μM α_3C	11
3. Absorption spectrum of α_3C from 200 to 800 nm	12
4. pH dependence of Lys	13
5. Simulated absorption spectra for Gly dimer and tetramer	14
6. Computed spectra for Gly and Glu with different backbone capping strategies	15
7. Transitions in computed spectra of Gly/Lys/Glu arising from capping groups	16
8. Decomposition of Lowest Energy Transitions for Gly, Lys, Glu	17
9. Movie: Lys-Lys sidechain interactions mediated by water molecules	18
10. Movie: Lys-Lys sidechain interactions mediated by Glu carboxylate groups	19
11. Interaction timescales for Lys-Lys and Lys-Glu sidechains in MD trajectory	20
12. Lys amino and Glu carboxylate group separations (DS and NN residue pairs)	21
13. Simulated absorption spectra and RDF for NN Lys-Lys, Glu-Glu, and Lys-Glu pairs	22
14. Decomposition of Lowest Energy Transitions (DS Lys-Lys, Glu-Glu, Lys-Glu Pairs)	23
15. Assignment of CT vs. Non-CT transitions for Lys-Lys and Glu-Glu spectra	24
16. HOMO-LUMO gaps for Lys/Glu monomers and DS dimers	25
17. Simulated absorption spectra for Glu-Glu and Lys-Glu dimers with explicit water	26
18. Simulated absorption spectra for singly charged Lys-Glu dimers	27
19. Simulated absorption spectra for Lys-Ala, Lys-Val, Lys-Ile, Lys-Leu, and Lys-Cys	28

S1 Details of Experimental and Computational Methods

S1.1 Search and Selection of α_3C for Experimental and Computational Studies:

List of all available PDB codes were acquired from RCSB Protein Data Bank (www.rcsb.org) and FASTA text files corresponding to them were downloaded. The number of different amino acids in each polypeptide chain of each of the FASTA text files was counted using an in-house computer application compiled in C programming language. The polypeptide chains that did not contain any aromatic amino acids (Trp, Tyr and Phe) but contained two or more Lys residues were selected. For these polypeptides PDB files with atomic coordinates were downloaded. Mutual distance(s) between the ε-Nitrogen atoms of different Lys residues within a polypeptide were then calculated using an in-house computer application compiled in C programming language. A cutoff of 10 Å was further applied to in order to shortlist those polypeptides where Lys side chains were in close proximity. Finally, 2-Mercaptoethanol-α_3C protein (PDB ID: 2LXY), was selected for further studies based on high content and close proximity of Lys residues in it. The α_3C protein (Figure 1a) contains 17 Lys residues. Out of these, 14 Lys pairs are within 10 Å distance.

S1.2 Data for Expression and Purification of α_3C

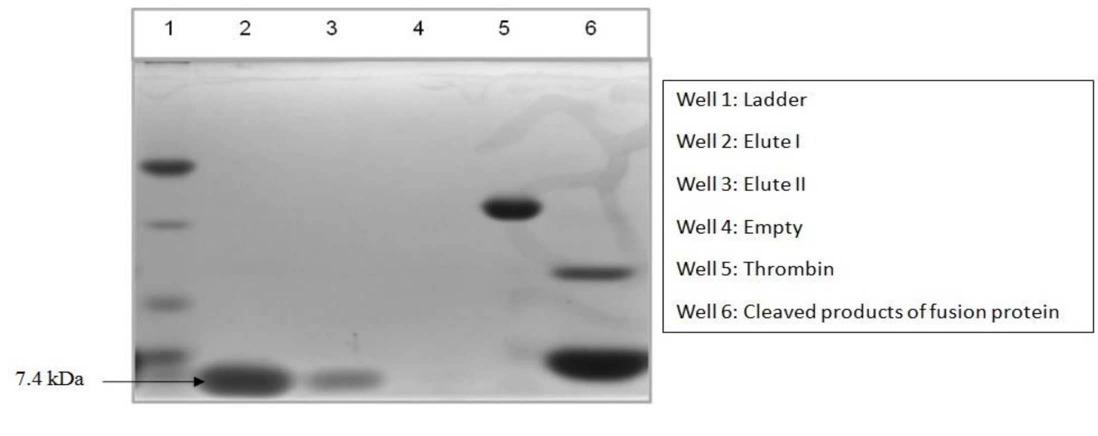

Figure S1a 15 % SDS-PAGE showing a single band at 7.4 kDa for the purified α_3C protein

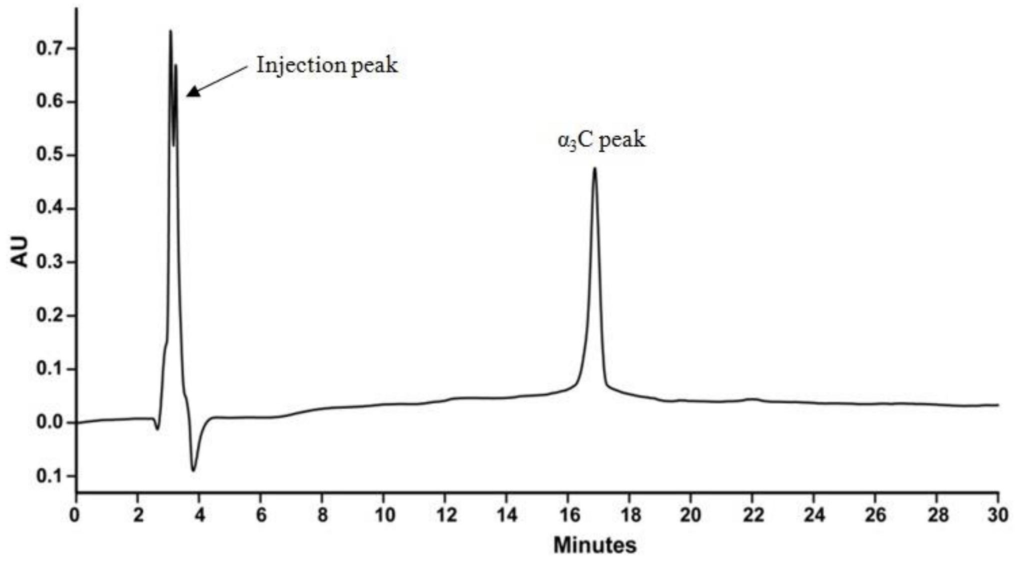

Figure S1b Reverse phase HPLC profile for α_3C

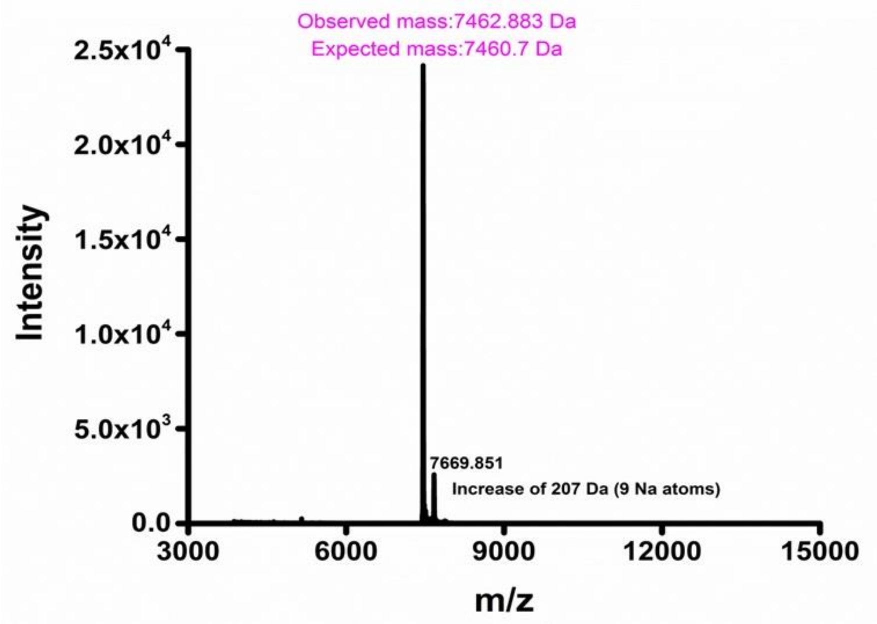

Figure S1c Mass spectrum of α_3C

S1.3 Solid Phase Peptide synthesis of Peptides Containing Lys (Synthesis)

Peptides with varying distance between the Lys residues were synthesized by standard Fmoc/tertiary-Butyl orthogonal protection strategy using solid phase peptide synthesis. The syntheses were performed manually on a Stuart blood tube rotator.

Peptides were synthesized such that each peptide had two Lys residues while the distance between them in the sequence kept on increasing.

Each step for peptide synthesis is described in detail below.

Unless stated specifically all reactions were carried out at room temperature.

STEP I: Swelling of Resin

a) 100 mg of Rink amide resin (loading 1.1 mmol/g) was soaked in 2 mL of Dichloromethane (DCM) for swelling.
b) DCM was then replaced with dimethylformamide (DMF) and the resin was further allowed to swell for another 1 hour.

STEP II: Deprotection of Fmoc group from the resin

a) 1.5 mL of 20% piperidine in DMF was added to the resin and the resin was washed 3 times for 7 minutes each.
b) After the deprotection was complete (about 21 minutes), the reaction column was drained and the resin was washed with (5 x 1.5 mL) portions of DMF for 1 minute each to remove piperidine.

STEP III: Amino Acid Coupling

a) In a small vial, 3 equivalents (98 mg) of Fmoc-AA (AA: Amino acid of interest) was pre-activated by combining it with 3.5 equivalents (170 mg) of BOP, 6 equivalents (85 mg) of DIPEA and 3 mL of DMF.
b) The contents were fully dissolved and then added to the activated resin.
c) The coupling was allowed to occur for 3 hours at room temperature.

d) Since the coupling of the first amino acid is often difficult, the above steps were repeated to ensure proper coupling of the first amino acid.

STEP IV: Washing

a) The resin containing the peptide was washed thoroughly with (5 x 1.5 mL) portions of DMF for 1 minute each to remove unbound amino acid.

STEP V: Kaiser Test

The Kaiser test is a qualitative test performed to monitor completeness of amino acid coupling in Solid Phase Peptide Synthesis. The test is based on the reaction of ninhydrin with primary amines, which gives a characteristic dark blue color. The test is used to monitor the presence of free amine after deprotection (dark blue color) and the completeness of the amino acid coupling step (yellow color).

a) Few resin beads were taken in a fusion tube.
b) Kaiser A solution (5% Ninhydrin in ethanol) and Kaiser B solution (Pinch of KCN in Pyridine: Ethanol solution, 80:20) was added in the tube.
c) The contents were heated for 5 minutes up to 80-85 ˚C on a sand bath.
d) The Kaiser test was negative as there was no blue color rather yellowish color was observed.

STEP VI: Capping

Apparently there was no free amine present in the resin, still capping of the resin was performed to make sure there were no free amine was present. Before performing this step the resin was washed with DCM (3 x 1.5 mL) for 1 minute each. Then,

a) Three fold molar excess of each; acetic anhydride and N-methyl imidazole were dissolved in DCM and added to the resin.
b) The reaction was kept for 40 minutes at room temperature.
c) The solution was filtered and the peptide resin was washed alternately with DCM followed by DMF.

STEP VII: Deprotection of amino acid (Fmoc cleavage from the amino acid)

a) 1.5 mL of 20% piperidine in DMF was added to the resin and the resin was washed 3 times for 7 minute each.
b) After the deprotection was complete (about 21 minutes), the reaction column was drained and the resin was washed with (5 x 1.5 mL) portions of DMF for 1 minute each to remove piperidine

Steps III through VII were repeated until the desired peptide sequence was synthesized on the resin. The last step was to cleave the peptide from the resin and collect it.

STEP VIII: Final peptide cleavage from the resin

a) After final washing with DMF, the resin was washed with (6 x 1.5 mL) portions of DCM for 1 minute each.
b) 2 mL of cleavage cocktail (TFA: DCM; 8.5: 1.5) was added to resin and the final cleavage was allowed to occur for 3 hours.

STEP IX: Peptide Precipitation

a) The contents were transferred to a tube containing 10 mL of chilled diethyl ether.
b) The contents were then centrifuged at 4000 rpm for about 10 minutes.
c) Supernatant was discarded and the pellet was stored at -20 ˚C

STEP X: Peptide purification and characterization

The synthesized crude peptides were dissolved in water/acetonitrile (2:1) and purified by Waters 600E RP-HPLC with a flow rate of 4 mL/ minute. Binary solvent system was used: solvent A (0.1 % TFA in water) and solvent B (0.1 % TFA in acetonitrile). C18-µ Bondapak column and a Waters 2489 UV detector were used with the detection at 214 nm. A total run time of 20 minutes and linear gradient was employed, as mentioned in the HPLC profiles. The purified peptides were characterized by mass spectrometry. A very small amount each peptide was dissolved in water/acetonitrile (2:1) solvent and then filtered with 0.2 µm filter. The mass of each peptide was then recorded in a Mass

Spectrometer. (Make: Agilent, Q-TOF 6500) in ESI positive mode. All the peptides were later lyophilized and stored at -20 ˚C for further studies.

S1.4 Solid Phase Peptide synthesis of Peptides Containing Lys (Characterization)

Peptide 1: NH_2-G-**K**-**K**-G-$CONH_2$

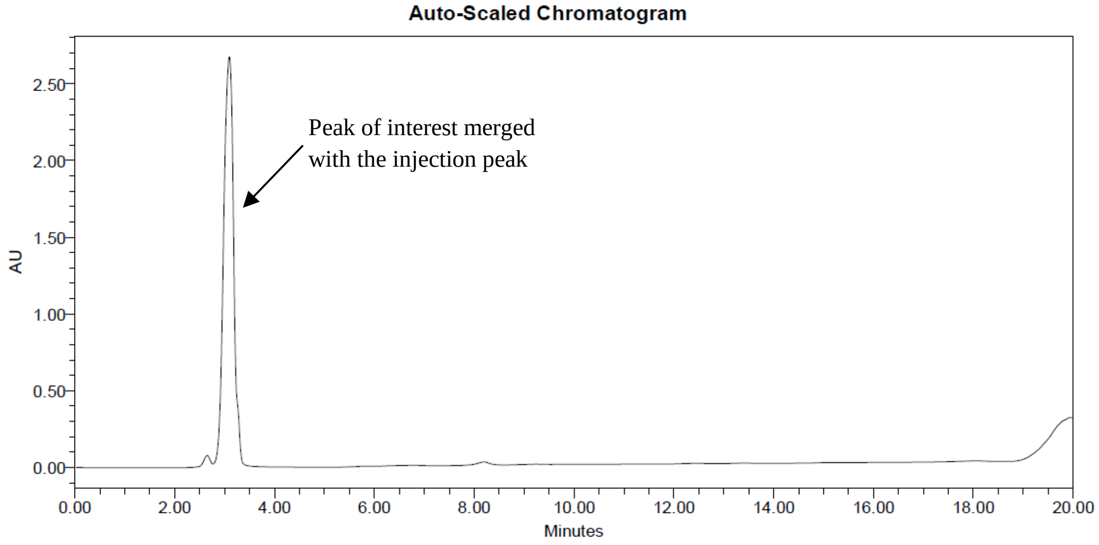

Figure S1d: HPLC profile of Peptide 1.Gradient: 0-15 min 0-10 % CH_3CN, 15-18 min 10-100% CH_3CN and 18-20 min 100% CH_3CN. Retention time: 3.1 minutes

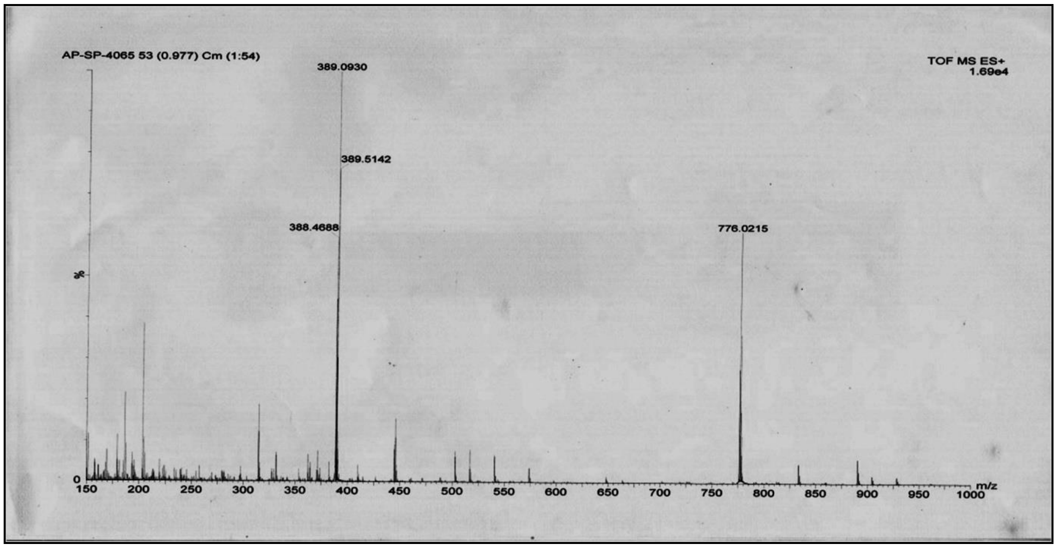

Figure S1e: Mass spectrum (ESI-MS) of Peptide 1. Calculated mass for $C_{16}H_{34}N_7O_4$ is 388.48 Da $[M+H]^+$, observed 389.09 Da [M+H]

Peptide 2: NH_2-G-**K**-A-**K**-G-$CONH_2$

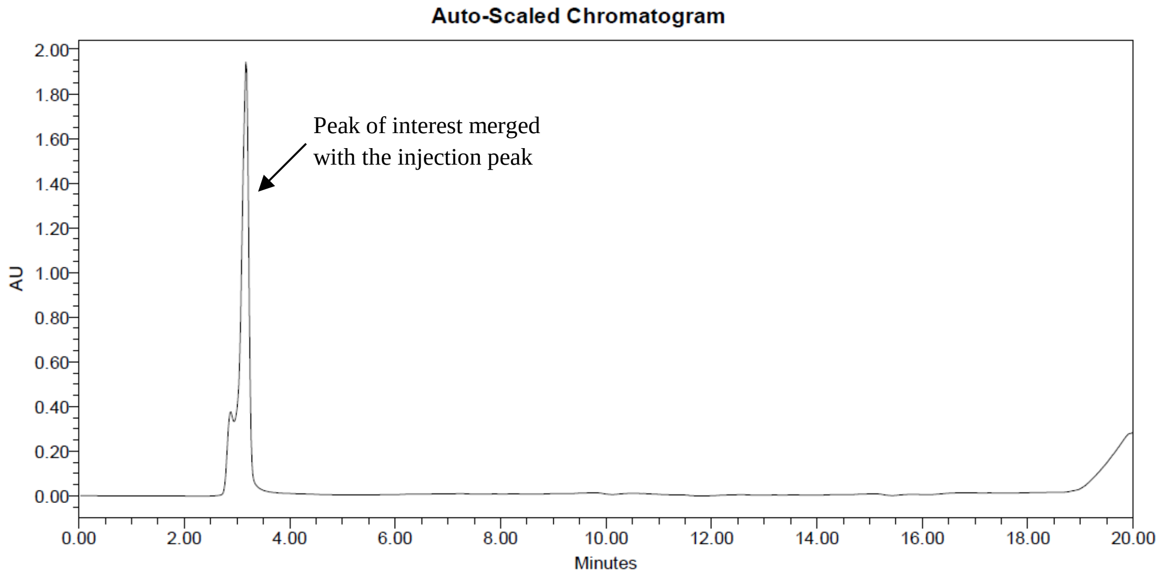

Figure S1f: HPLC profile of Peptide 2.Gradient: 0-15 min 0-10 % CH_3CN, 15-18 min 10-100% CH_3CN and 18-20 min 100% CH_3CN. Retention time: 3.1 minutes

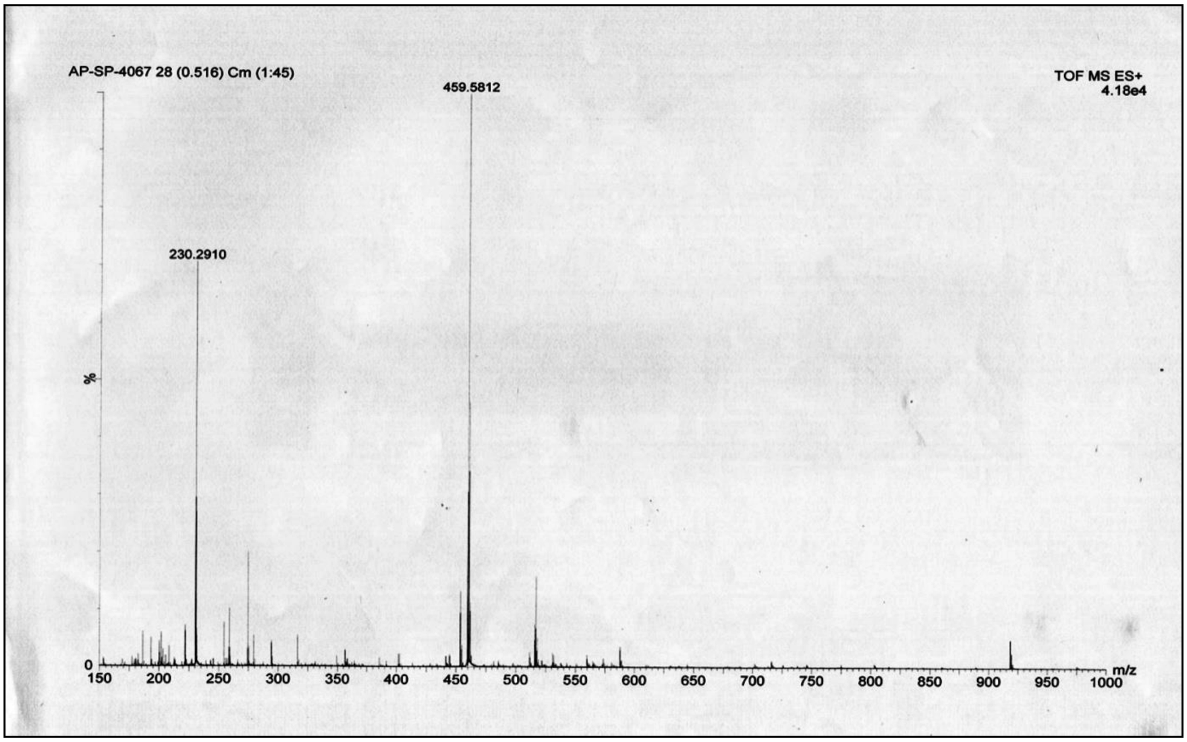

Figure S1g: Mass spectrum (ESI-MS) of Peptide 2. Calculated mass for $C_{19}H_{39}N_8O_5$ is 459.56 Da $[M+H]^+$, observed 459.58 Da $[M+H]^+$

Peptide 3: NH_2-G-**K**-A-A-**K**-G-$CONH_2$

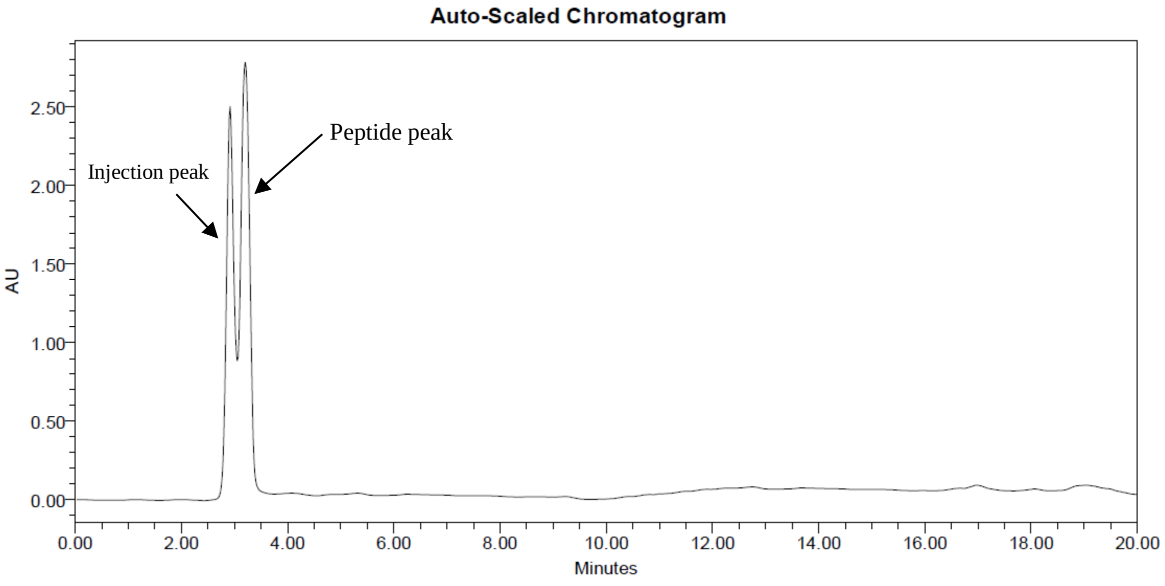

Figure S1h: HPLC profile of Peptide 3. Gradient: 0-15 min 0-10 % CH_3CN, 15-18 min 10-100% CH_3CN and 18-20 min 100% CH_3CN. Retention time: 3.3 minutes

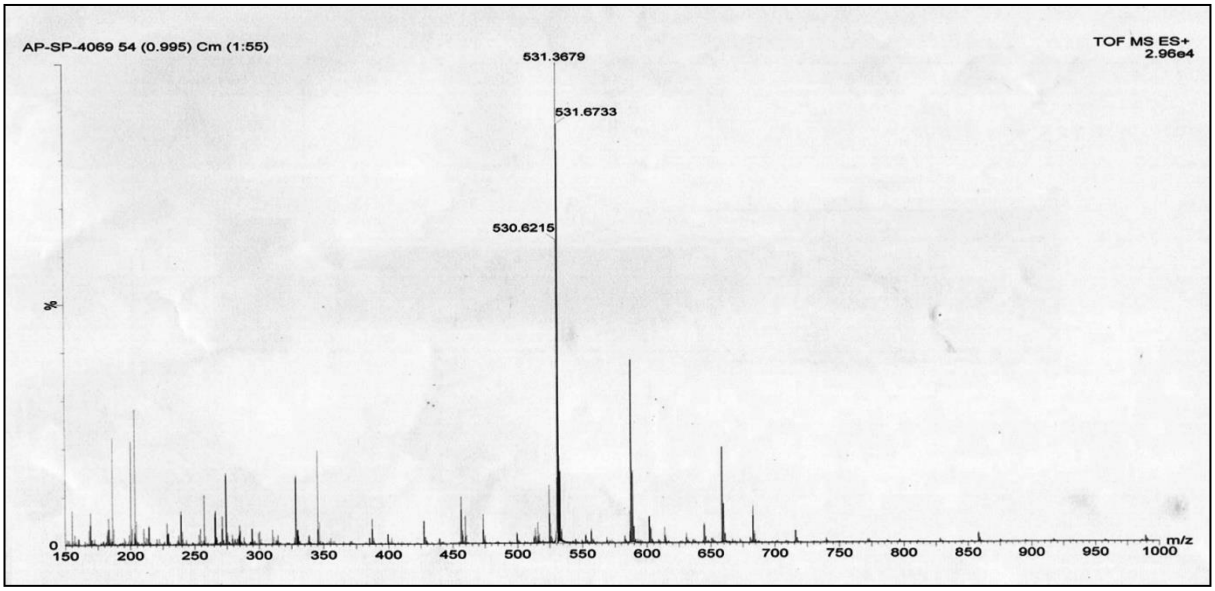

Figure S1i: Mass spectrum (ESI-MS) of Peptide 3. Calculated mass for $C_{22}H_{44}N_9O_6$ is 530.63 Da $[M+H]^+$, observed 531.36 Da $[M+H]^+$

S1.5 Equilibration Protocol for Molecular Dynamics Simulations:

We carried out MD simulations on fully solvated atomistic models of the α_3C protein. The initial structure used in the simulations was an NMR derived structure (PDB Code: 2LXY) captured with Mercaptophenol ligated at the C32 site. The ligand was removed during processing to carry out simulations of Mercaptophenol free α_3C. The protein was solvated (TIP3P water model) inside a rectangular water box of dimensions ~67x56x60Å^3 and neutralized by adding 2 Cl^- ions. Our simulations employed periodic boundary conditions with the Particle Mesh Ewald method for describing electrostatic interactions. The van der Waals forces were calculated with the use of a switching function with 10 Å switching distance and 12 Å cutoff. The equilibration protocol comprised of an initial 10000 step energy minimization step, followed by gradual heating from 0 K to 300 K over 50 ps, and thermal equilibration at 300 K for another 50 ps. These steps were initially performed with the protein heavy atoms fixed (unconstrained hydrogens, ions and waters) and then with the harmonic constraint of 25 kcal/mol/Å^2 on the protein heavy atoms. We then carried out constant pressure and temperature (NPT) equilibrations with a harmonic constraint of 25 kcal/mol/Å^2 on the protein heavy atoms for 150 ps to stabilise the density of the system at 1 atm pressure and temperature 300 K. The pressure of the system was maintained by the Nose–Hoover method in combination with Langevin dynamics to control the temperature of the system. The NPT protocol was repeated 3 more times with harmonic constraints set to 12, 6 and 3 kcal/mol/Å^2. Then an unconstrained 200 ps NPT (NPT-free) run was performed at 300 K to equilibrate the system. Finally a 110 ns MD NPT production run was carried out generating snapshots at interval of 2 ps.

S2 Amino acid concentrations in 105 µM α_3C

Sl. No.	Amino Acid	Count in α_3C	Concentration (µM)
1	Alanine (A)	3	315
2	Arginine (R)	2	210
3	Asparagine (N)	0	0
4	Aspartic Acid (D)	0	0
5	Cysteine (C)	1	105
6	Glutamine (Q)	0	0
7	Glutamic acid (E)	17	1785
8	Glycine (G)	9	945
9	Histidine (H)	0	0
10	Isoleucine (I)	3	315
11	Leucine (L)	8	840
12	Lysine (K)	17	1785
13	Methionine (M)	0	0
14	Phenylalanine (F)	0	0
15	Proline (P)	0	0
16	Serine (S)	1	105
17	Threonine (T)	0	0
18	Tryptophan (W)	0	0
19	Tyrosine (Y)	0	0
20	Valine (V)	6	630
**Total no. of amino acids**		**67**	N/A

Table S2. Population of constituent amino acids in α_3C sequence and their individual concentrations in 105 µM of protein.

S3 Absorption spectrum of α_3C from 200 to 800 nm

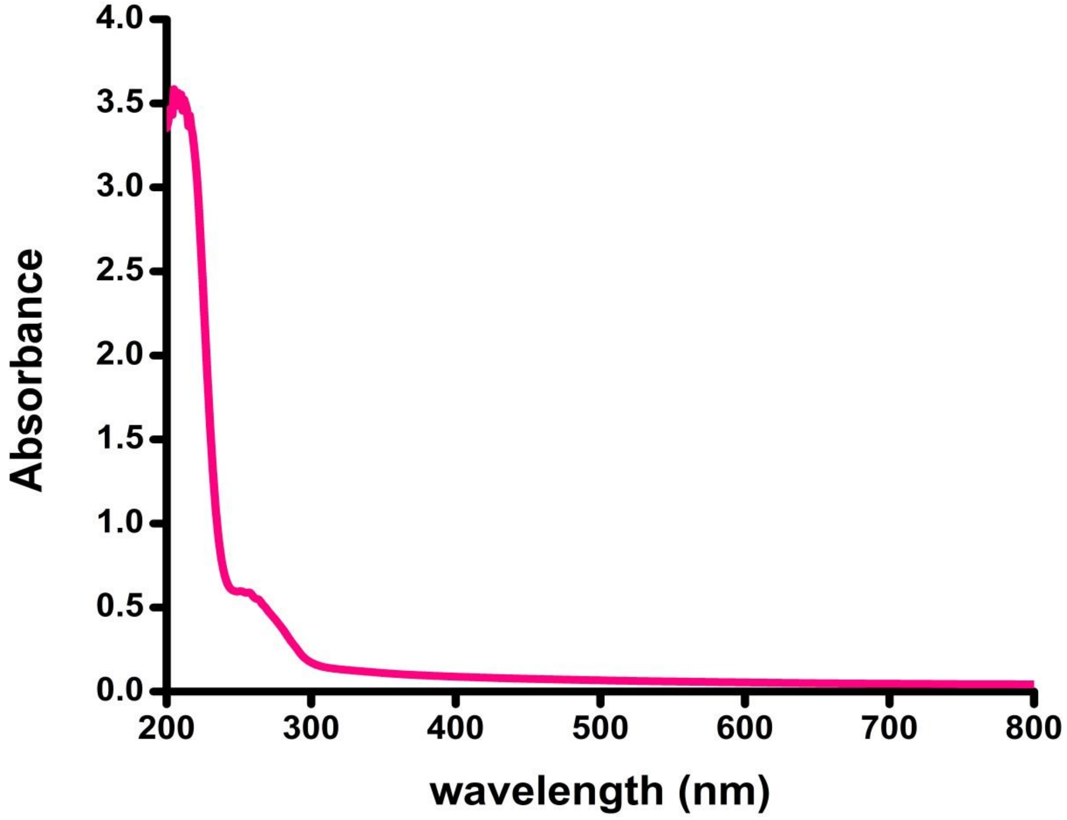

Figure S3. Absorption spectrum (200-800 nm) for α_3C (85 µM) in deionized water

S4 pH dependence of Lys

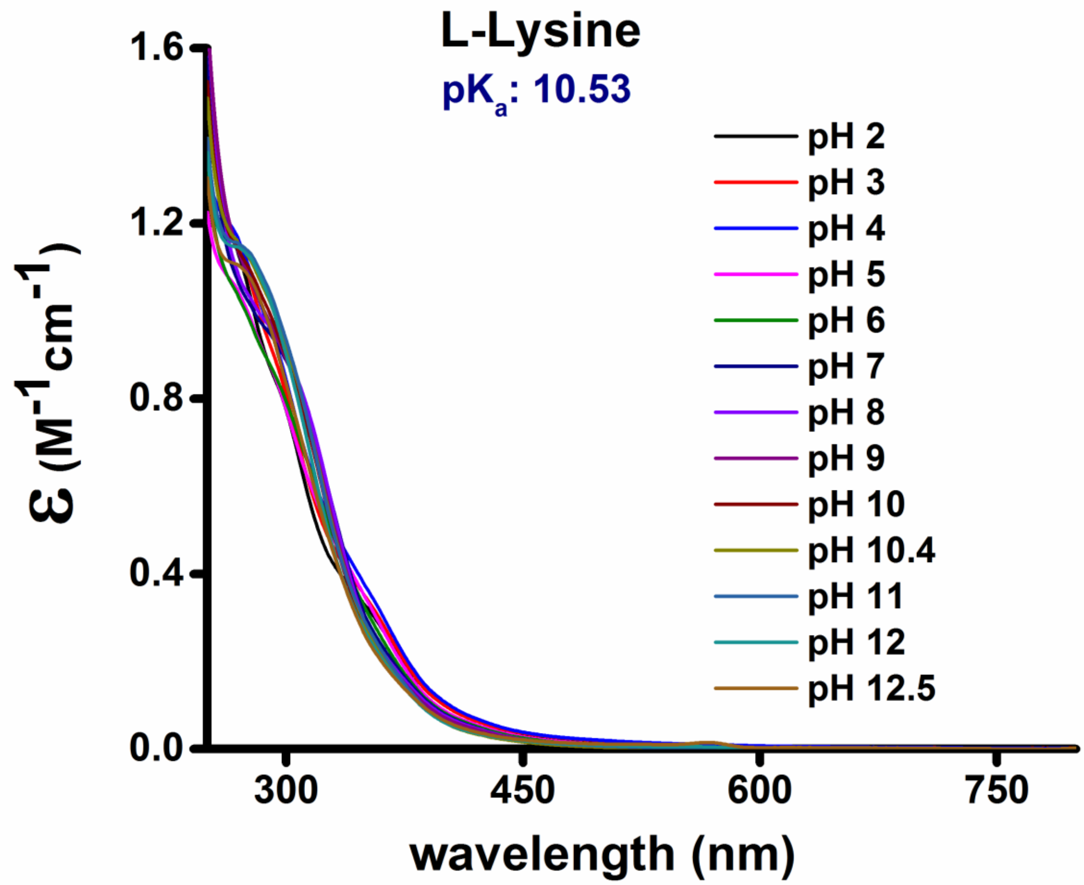

Figure S4. Absorption spectra of Lys (1 M) in deionized water at different pH values.

S5 Simulated absorption spectra for Gly dimer and tetramer

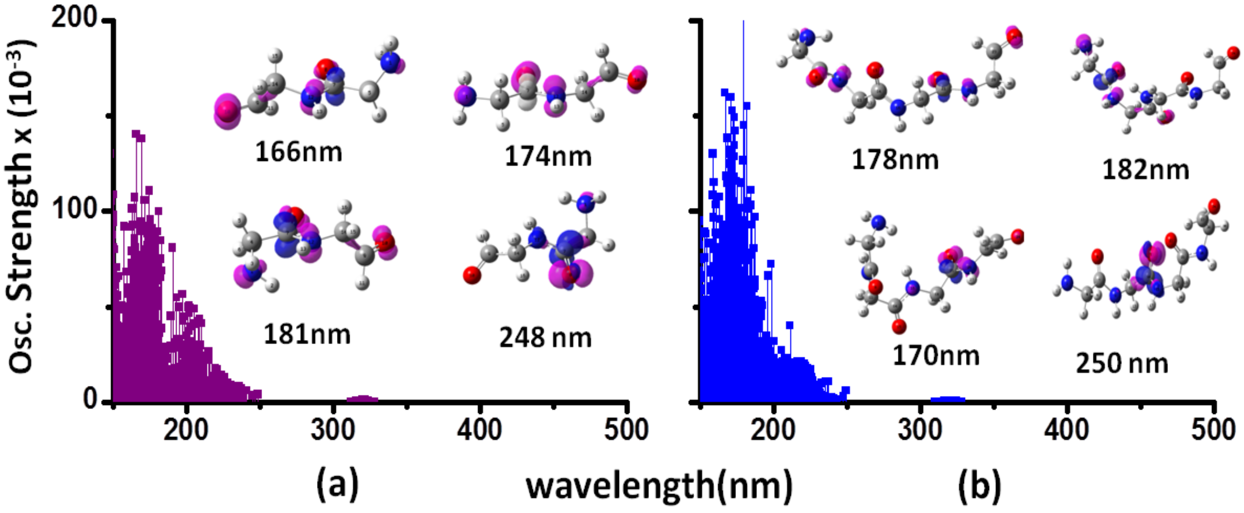

Figure S5. Simulated absorption spectra (wavelength vs. oscillator strength) of (a) Gly dimer, and (b) Gly tetramer. Each panel shows difference density plots (isovalue 0.01) visualizing hole (pink) and electron (blue) density localization on the amino acid fragments for the red most transitions in the spectra and for backbone CT transitions between 165-185 nm. The weak transitions above 300 nm are spurious transitions introduced due to the capping groups; see Fig S6 for more examples.

S6 Computed spectra for Gly and Glu with different backbone capping strategies

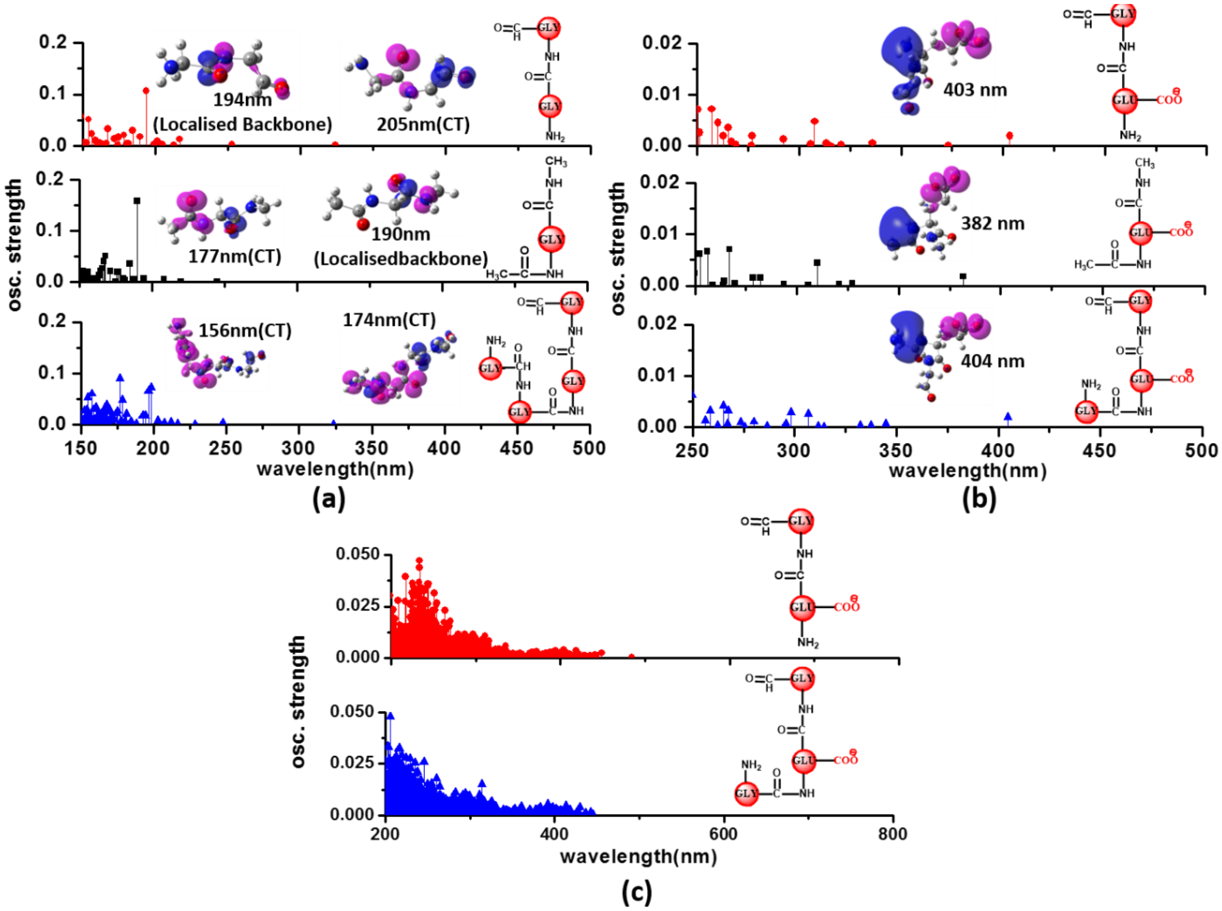

Figure S6. (a) Comparison of spectra from a single representative structure for Gly with different backbone capping strategies: (top panel) model used in the present study, (middle panel) extended backbone with methyl group capping, and (bottom panel) Gly tetramer with capping strategy in the present study. Representative difference density plots (isovalue 0.01) visualizing hole (pink) and electron (blue) density localization on the amino acid fragments are shown as insets for π-π^* backbone transitions around 190 nm (Oscillator strengths f ~ 0.1) and CT transitions at higher energies between 150-180 nm. (b) Comparison of spectra from a single representative structure for Glu with different backbone capping strategies: (top panel) model used in the present study, (middle panel) extended backbone with methyl group capping, and (bottom panel) extended backbone with Gly units appended symmetrically. Difference density plots for the red-most transitions are shown for each panel. (c) Comparison of the spectra for 100 conformations with the capping strategy used in the present study (top panel) and a model with symmetric Gly backbone extensions (bottom panel).

S7 Transitions in computed spectra of Gly/Lys/Glu arising from capping groups

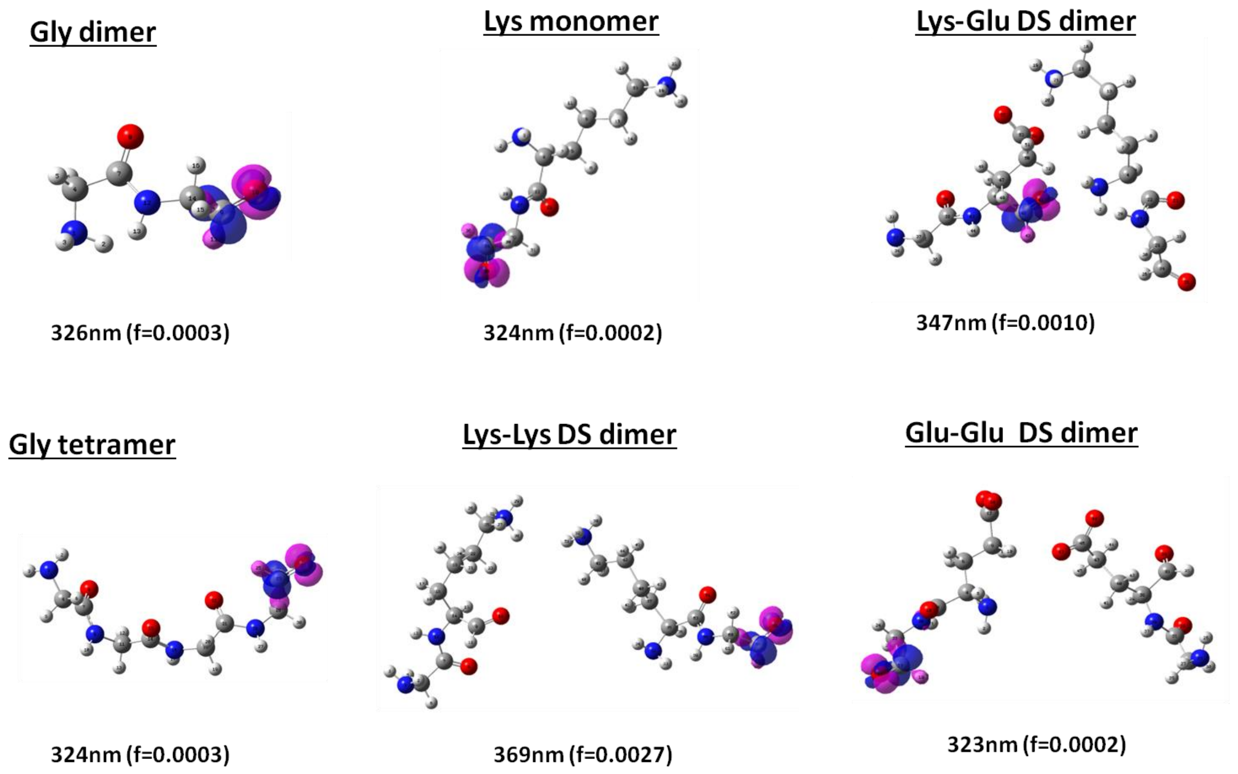

Figure S7. Weak transitions (non-CT) above 300 nm introduced by the truncation of the peptide backbone in our amino acid monomer and dimer models. The transitions are specific to the hydrogen capping strategy employed in our calculations and can be distinctly identified and separated from our characteristic CT transitions of interest.

S8 Decomposition of Lowest Energy Transitions for Gly, Lys, Glu

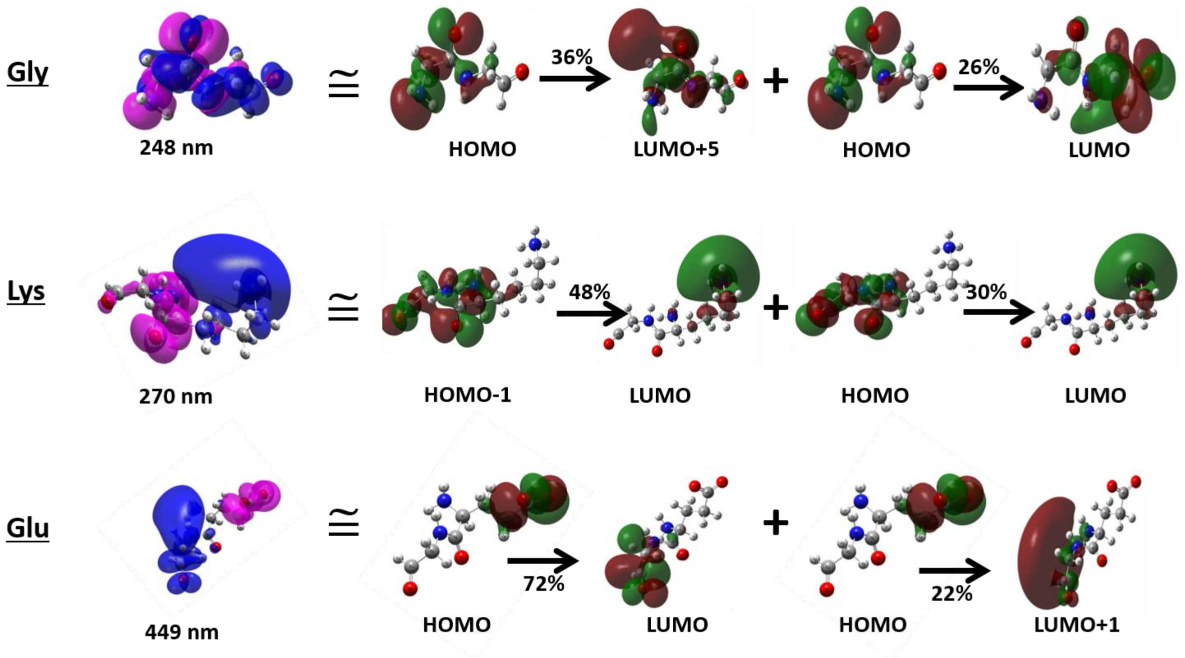

Figure S8: Top two dominant molecular orbital (MO) pair contributions for the transitions depicted in Figure 4 (main manuscript) for Gly, Lys, and Glu amino acids through difference density plots.

S9 Movie: Lys-Lys sidechain interactions mediated by water molecules

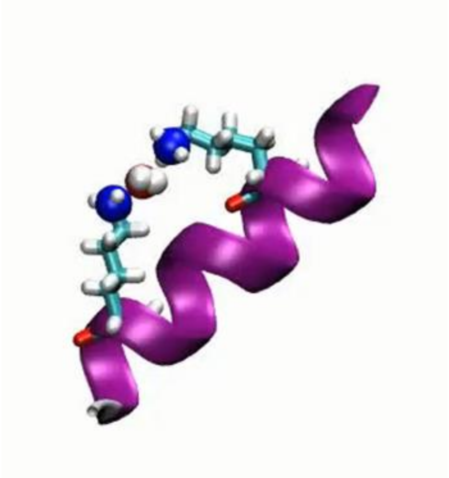

Movie-S9.mpg showing the interactions between the amino groups of two distally separated (DS) Lys residues mediated by water. Movie files have been uploaded separately as part of the supporting information.

S10 Movie: Lys-Lys sidechain interactions mediated by Glu carboxylate groups

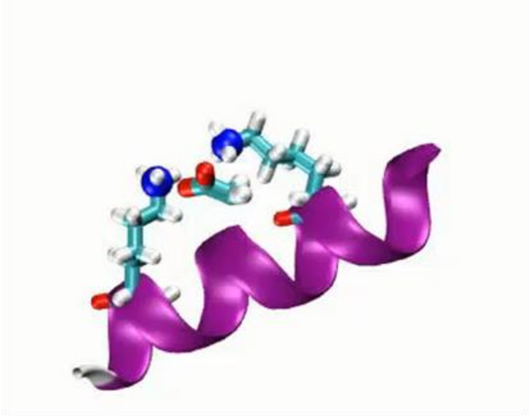

Movie-S10.mpg showing the interactions between the amino groups of two distally separated (DS) Lys residues mediated by a Glu carboxylate group. Movie files have been uploaded separately as part of the supporting information.

S11 Interaction timescales for Lys-Lys and Lys-Glu sidechains in MD trajectory

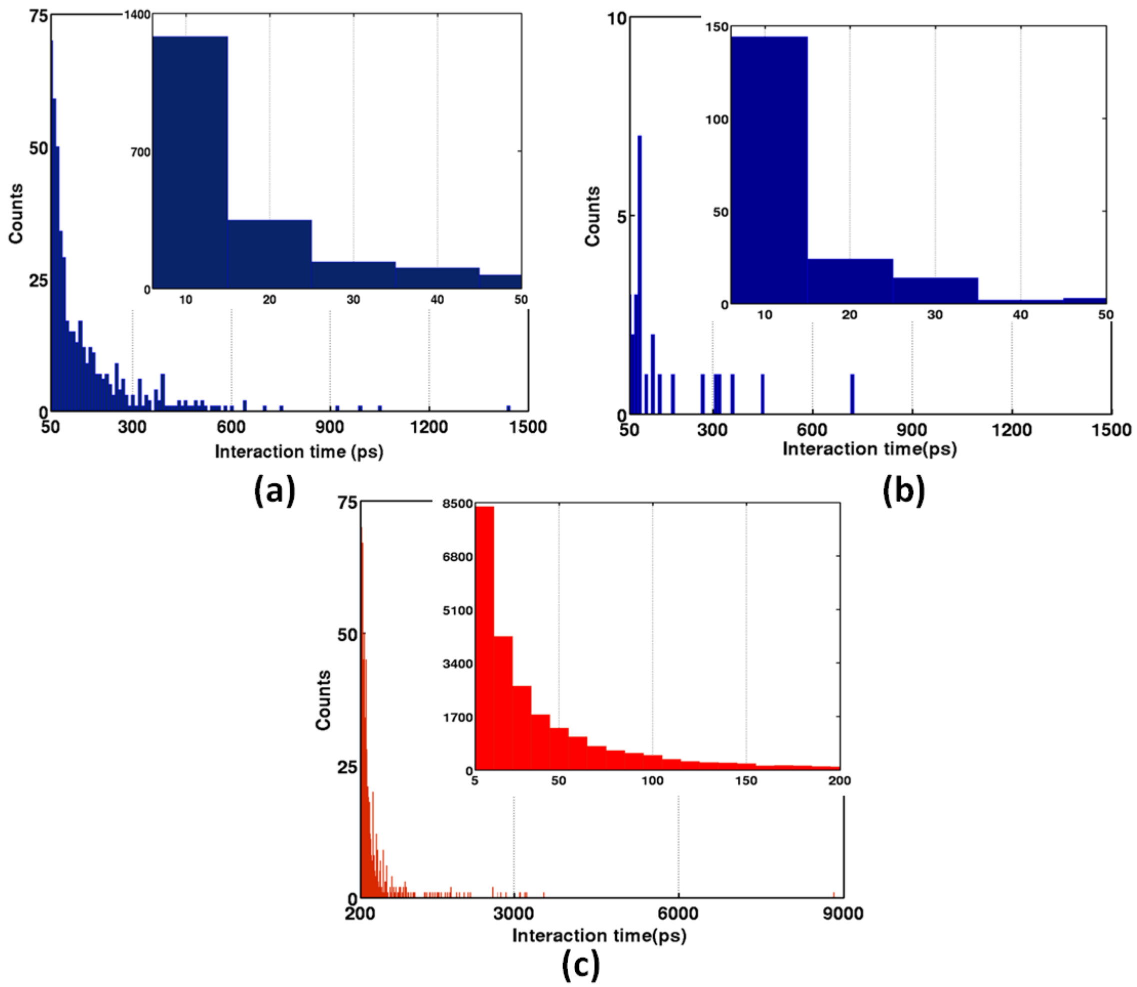

Figure S11. Histograms displaying interaction timescales for strongly interacting DS and NN Lys-Lys pairs (blue: a, b) and DS Lys-Glu pairs (red: c). Insets in each plot show the distributions at short timescales (up to 50 ps for Lys-Lys and up to 200 ps for Lys-Glu pairs). We do not find statistically significant instances of strongly interacting NN Lys-Glu pairs in our MD trajectories. For the Lys-Lys pairs considered here, N_A atom pairs are separated by 3-5.5 Å. For Lys-Glu pairs considered here Lys N_A atom and Glu C_C atoms are separated by 2-4 Å.

S12 Lys amino and Glu carboxylate group separations (DS and NN residue pairs)

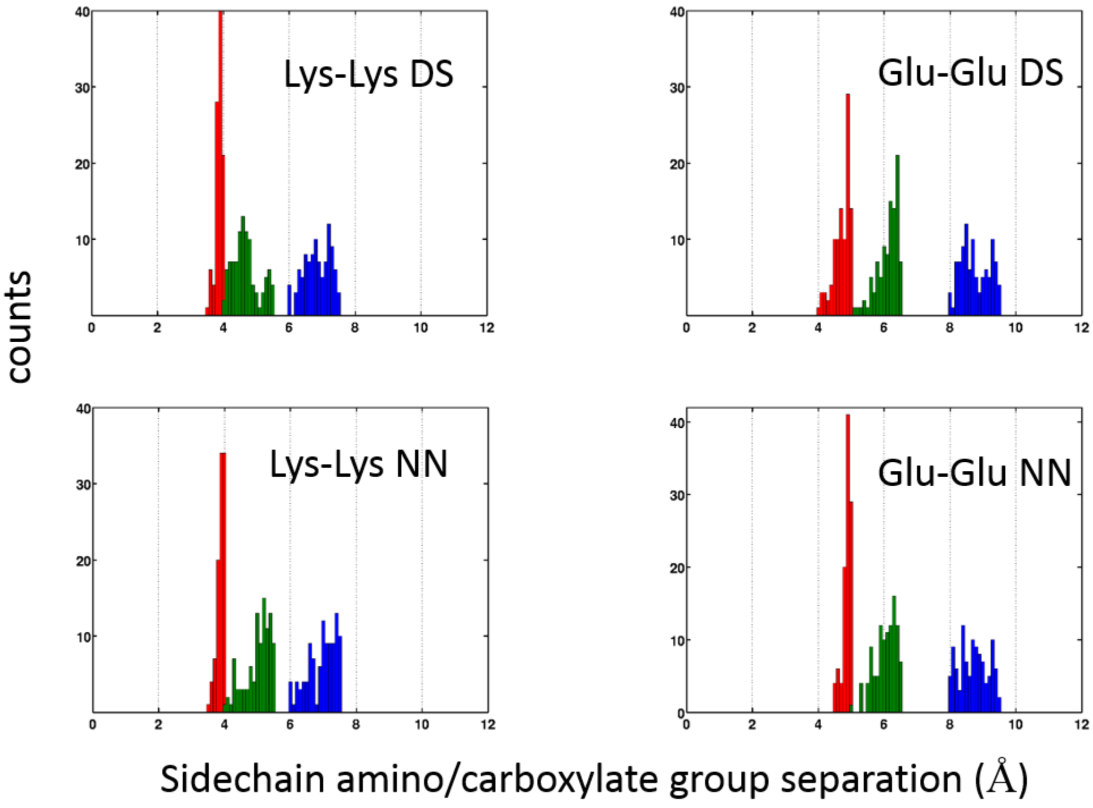

Figure S12. Histograms displaying interaction distances between Lys sidechains (N_A atoms) and Glu sidechains (C_C atoms). Top panels show interactions for distally separated (DS) residue pairs in the protein sequence. Bottom panels show interaction distances for nearest neighbour (NN) residue pairs in the protein sequence. The three colours represent strong (red), intermediate (green), and weak (blue) interactions as described in Figure 6 of the main manuscript.

S13 Simulated absorption spectra and RDF for NN Lys-Lys, Glu-Glu, and Lys-Glu pairs

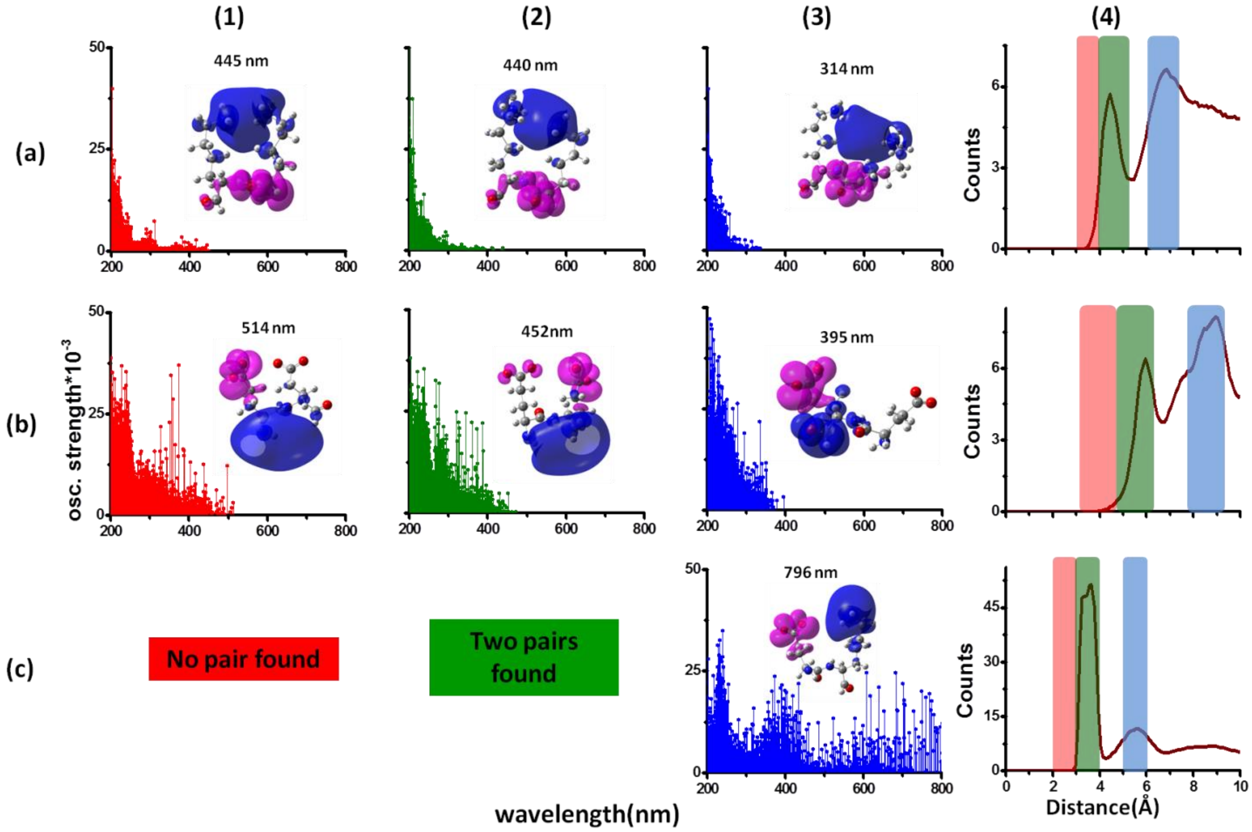

Figure S13. Simulated absorption spectra (wavelength vs. oscillator strength) of NN Lys pair for 3-4 (red), 4-5.5 (green) and 6-7.5 (blue) Å distance range and radial distribution function (RDF) plot of Lys N_A atoms (a1-a4); Simulated absorption spectra of NN Glu pairs for 3.5-5 (red), 5-6.5 (green) and 8-9.5 Å (blue) distance range and RDF of C_C atoms of Glu (b1-b4); Simulated spectra of NN Lys- Glu pairs for 5-6 Å (blue) distance range and RDF of Lys N_A and Glu C_C atoms (c3-c4). We do not find statistically significant number of NN Lys-Glu pairs exhibiting intermediate or strong interactions. Each absorption spectra panel (a1-a3, b1-b3, and c3) contains difference density plots showing hole (pink) and electron (blue) densities on Lys/Glu fragments for lowest energy transitions.

S14 Decomposition of Lowest Energy Transitions (DS Lys-Lys, Glu-Glu, Lys-Glu Pairs)

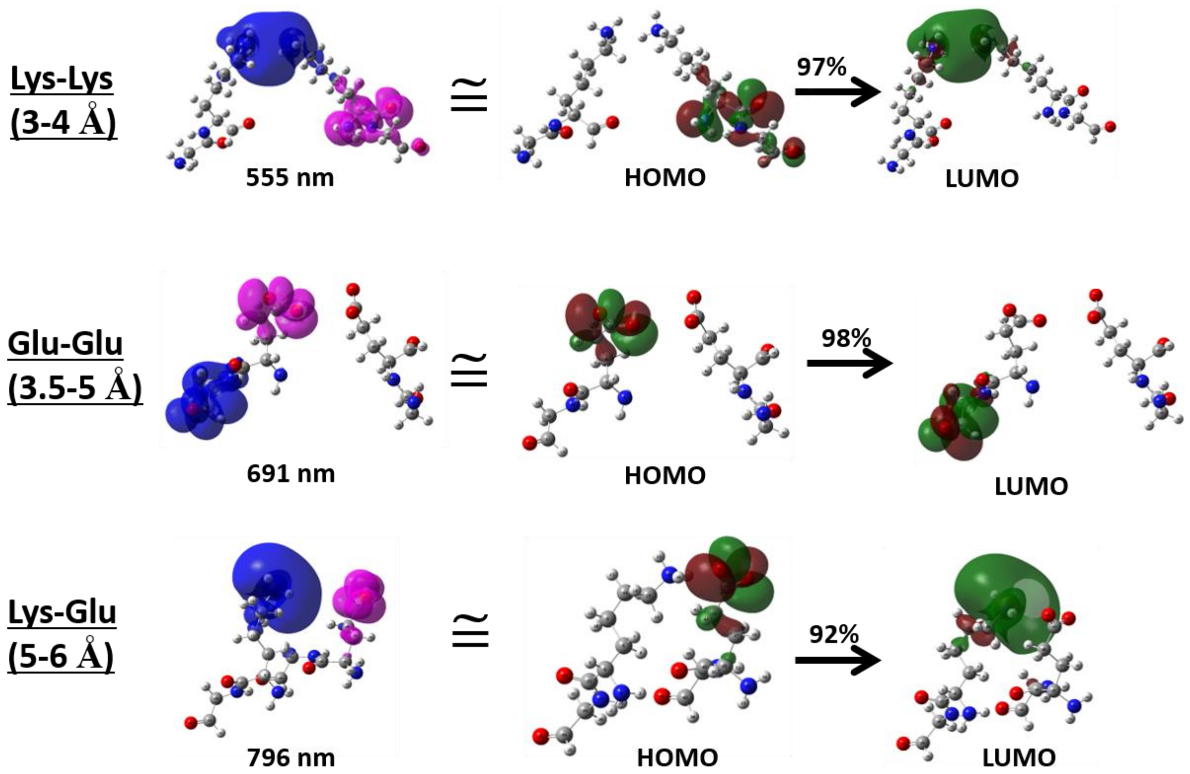

Figure S14: Dominant molecular orbital (MO) pair contributions for the transitions depicted in Figure 6 (main manuscript) through difference density plots.

S15 Assignment of CT vs. Non-CT transitions for Lys-Lys and Glu-Glu spectra

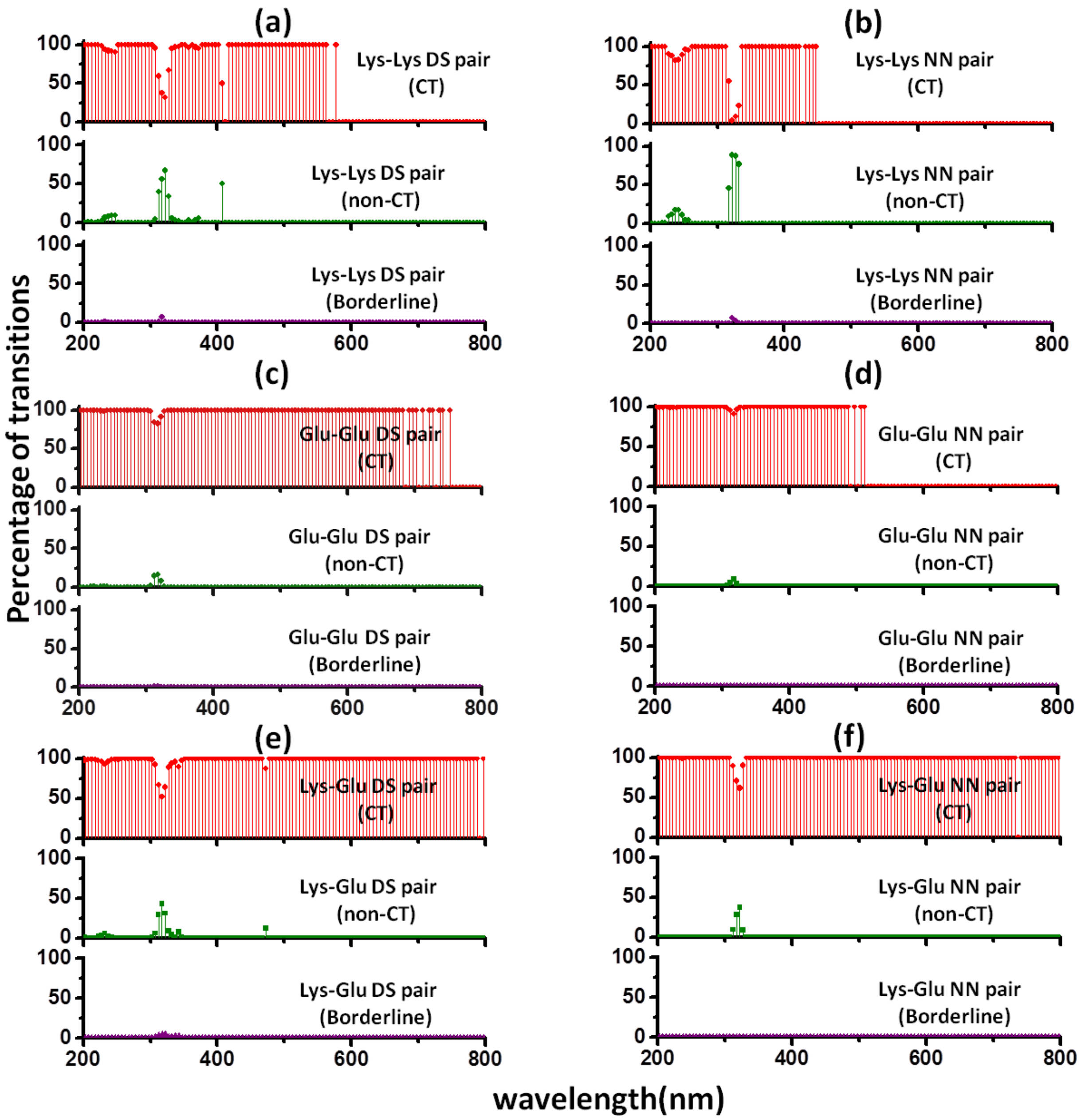

Figure S15. Percentage of CT (red) vs. non-CT (green) transitions as a function of wavelength for (a) DS Lys-Lys, (b) NN Lys-Lys, (c) DS Glu-Glu, (d) NN Glu-Glu, (e) DS Lys-Glu and (f) NN Lys-Glu pairs in strong interaction region. Transitions were binned with 5 nm increments. Transitions for which the charge separation measure Δr (equation 1 of main manuscript) was within 5 % of the threshold (2 Å) value were classified as borderline transitions.

S16 HOMO-LUMO gaps for Lys/Glu monomers and DS dimers

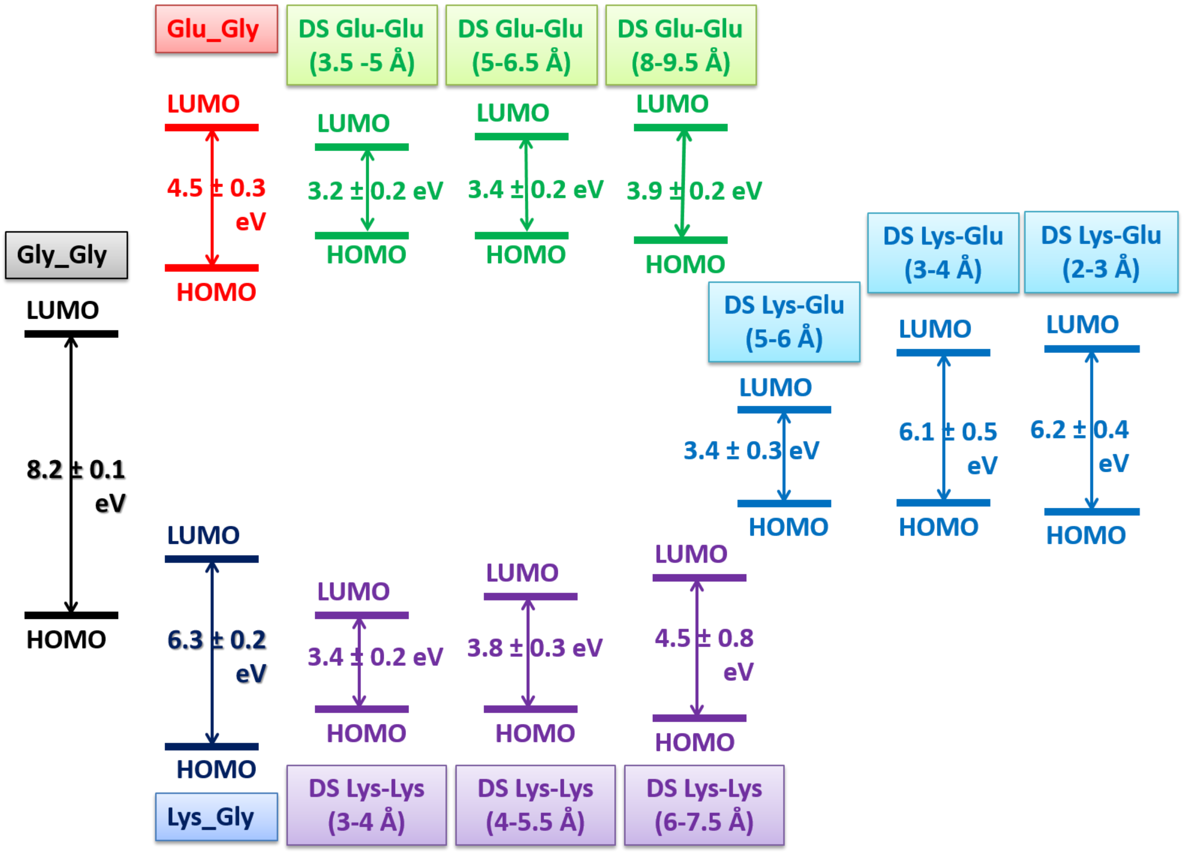

Figure S16. Trends in average ground state HOMO-LUMO gaps for monomer and dimer fragments along with their standard deviations from the 100 MD snapshots which were used to compute the spectra in Figure 4 and Figure 6 of the main manuscripts. We compare trends in HOMO-LUMO gaps for monomer vs dimer species, and for dimers as a function of distance between their charged amino/carboxylate groups.

S17 Simulated absorption spectra for Glu-Glu and Lys-Glu dimers with explicit water

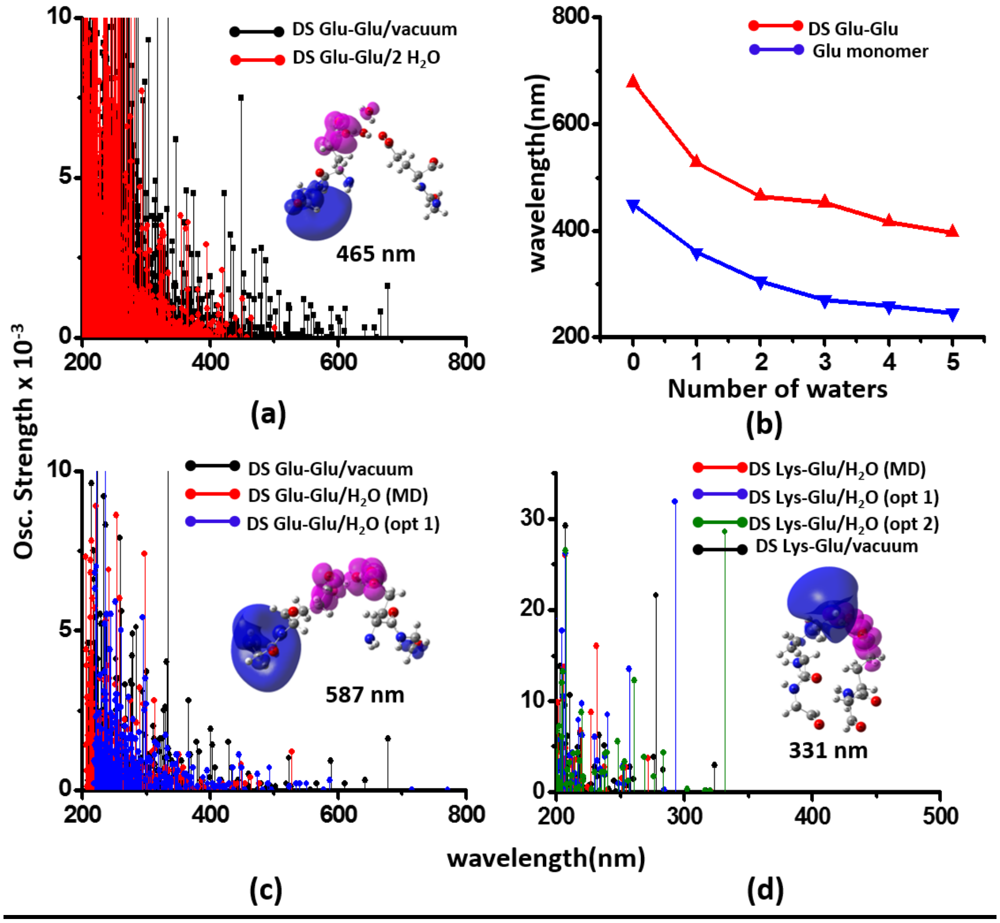

Figure S17. Modulation of DS Glu-Glu dimer (C_C-C_C distance 3.5-5Å) and DS Lys-Glu dimer (N_A-C_C distance 3.66 Å) spectra with inclusion of explicit waters: (a) DS Glu-Glu vacuum vs 2 waters (b) relative shifts in lowest energy transition wavelengths with number of explicit waters for Glu-Glu dimer and Glu monomer spectra, (c) Influence of water position (single explicit water) on the computed DS Glu-Glu spectral range. Panels a and b show results from spectra calculations on 10 representative conformations sampled from MD. In panel c (single MD snapshot: C_C- C_C distance is 4.33 Å) spectra were calculated for two positions of water oxygen relative to C_C of the Glu pairs: 1) MD snapshot (3.36 Å, 3.60 Å), opt 1 (2.75 Å, 3.12 Å). In panel d (single MD snapshot) spectra were calculated for two positions of water oxygen relative to N_A and C_C of the Lys-Glu pair respectively: MD snapshot (3.11 Å, 3.37 Å), opt 1 (2.47 Å, 2.50 Å), and opt 2 (2.10 Å ,2.12 Å). Different density plots of the lowest energy transitions for all calculations with explicit water are shown as insets.

S18 Simulated absorption spectra for singly charged Lys-Glu dimers

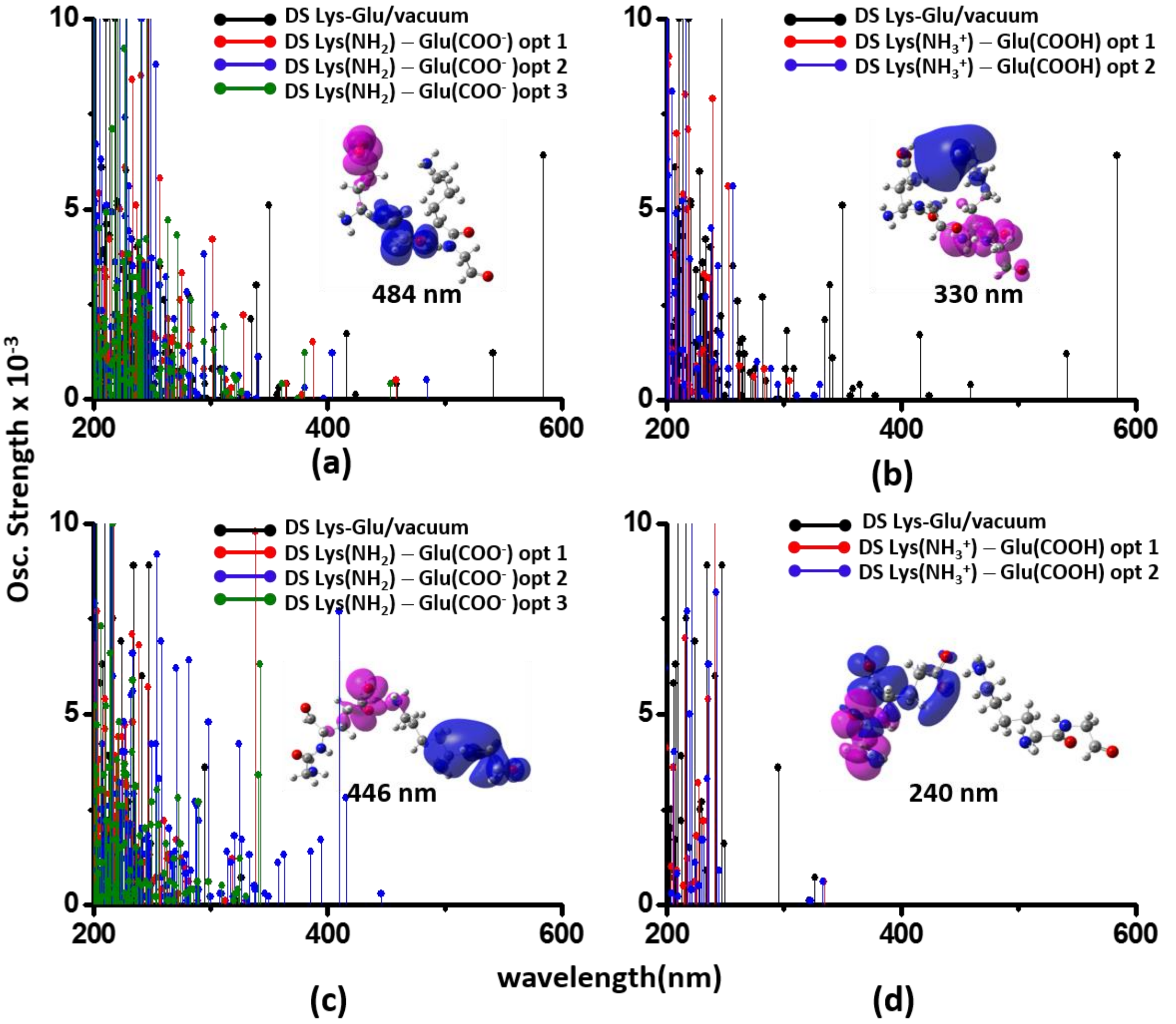

Figure S18. Modulation of DS Lys-Glu dimer absorption (single snapshot from MD) due to NH_3^+ deprotonation (panels a and c) and COO^- protonation (panels b and d) in the pair. In panels a and c, three data sets (opt 1, opt 2, and opt 3) corresponding to removal of different hydrogen atoms from the amino group are shown. In panels b and d, two data sets (opt 1 and opt 2) corresponding to the addition of hydrogen atoms to the two carboxylate oxygens are shown. Panels (a) and (b) represent a weakly interacting DS Lys-Glu pair (N_A – C_C distance 5.49 Å). Panels (c) and (d) represent a strongly interacting DS Lys-Glu pair (N_A – C_C distance 2.97 Å). Different density plots of the lowest energy transitions for deprotonated amino or protonated carboxy group calculations are shown as insets.

S19 Simulated absorption spectra for Lys-Ala, Lys-Val, Lys-Ile, Lys-Leu, and Lys-Cys

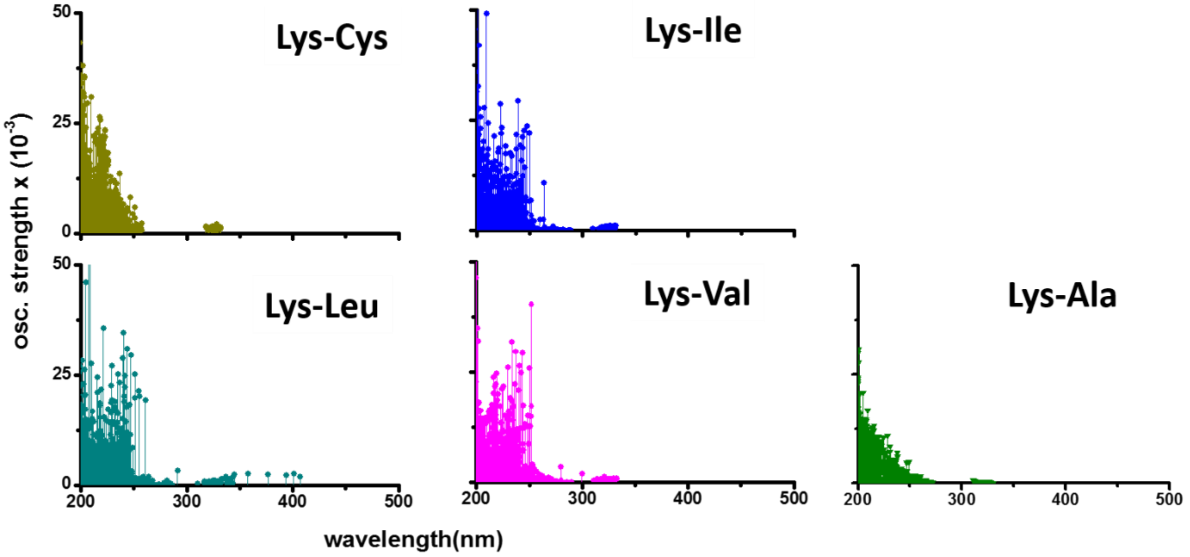

Figure S19. Simulated absorption spectra of all NN Lys-AAA dimers, where AAA=Leu, Cys, Ile, Val, and Ala. Together with the Lys-Lys, Lys-Glu, and Glu-Glu pairs considered in Figure S13, these dimers represent all NN amino acid dimers containing Lys which are present in α_3C.
